# A longitudinal study on the impact of the TyG Index and TG/HDL-C ratio on the risk of type 2 diabetes in Chinese patients with prediabetes

**DOI:** 10.1186/s12944-024-02239-1

**Published:** 2024-08-22

**Authors:** Bo Chen, Jingjing Zeng, Menglin Fan, Qiqi You, Chenyue Wang, Ke Wang, Minghui Qin, Shaoyong Xu

**Affiliations:** 1 Department of Endocrinology, Xiangyang Central Hospital, Affiliated Hospital of Hubei University of Arts and Science, Xiangyang, Hubei China; 2 Center for Clinical Evidence-Based and Translational Medicine, Xiangyang Central Hospital, Affiliated Hospital of Hubei University of Arts and Science, Xiangyang, Hubei China; 3 Department of Preventive Medicine, Medical College, Hubei University of Arts and Science, Xiangyang, 441053 China; 4 Department of Traditional Chinese Medicine, Xiangyang Central Hospital, Affiliated Hospital of Hubei University of Art and Science, Xiangyang, Hubei China

**Keywords:** TyG, Diabetes, TG/HDL-C ratio, Area under curve

## Abstract

**Objective:**

To elucidate the impact and predictive value of the Triglyceride Glucose Index (TyG) and the ratio of Triglycerides to High-Density Lipoprotein Cholesterol (TG/HDL-C) in identifying the risk of diabetes progression in Chinese individuals with prediabetes.

**Methods:**

This longitudinal study enrolled 15,012 prediabetic adults from the Rich Healthcare Group between 2010 and 2016. Diabetes was defined as self-reported diabetes or a fasting glucose level ≥ 7.0 mmol/L. The Cox proportional hazards models was utilized to assess the relationship between the two indices and the risk of developing diabetes. The predictive efficacy of the two markers was gauged by the area under the curve (AUC).

**Results:**

Over a median follow-up period of 2.87 years, 1,730 (11.5%) prediabetic participants developed diabetes. The adjusted hazard ratios for the top quartile of the TyG index and the TG/HDL-C ratio were 2.03 (95% confidence interval [CI]: 1.71–2.40) and 2.59 (95% CI: 2.20–3.05), respectively, compared to the lowest quartile. A significant trend of increasing diabetes risk with higher quartiles of both indices was observed. The AUC for the adjusted prediction model for prediabetes-to-diabetes transition was 0.726 for the TyG index and 0.710 for the TG/HDL-C ratio. The difference in AUCs was statistically significant (*P* = 0.03).

**Conclusions:**

The baseline TyG index or TG/HDL-C ratio was significantly associated with an increased risk of diabetes in prediabetic individuals. The TyG index demonstrated superior predictive accuracy, underscoring its importance in preventing diabetes in prediabetic individuals.

**Supplementary Information:**

The online version contains supplementary material available at 10.1186/s12944-024-02239-1.

## Introduction

Prediabetes is a critical health state in which blood glucose levels are higher than typical but do not reach the thresholds required for type 2 diabetes mellitus (T2DM) [1]. The prevalence of prediabetes is increasing globally, with projections estimating it will affect one billion individuals by 2045 [2, 3]. Each year, 5–10% of those with prediabetes progress to T2DM. Prediabetes is linked to an increased risk of macrovascular and microvascular complications, such as stroke, peripheral arterial disease, myocardial infarction and retinopathy, neuropathy and nephropathy [3–5]. Additionally, prediabetes is linked to an elevated risk of all-cause and cardiovascular mortality [6]. Despite these risks, the majority of individuals with prediabetes remain unaware of their metabolic condition, highlighting the critical need for early detection and intervention of modifiable factors.

Insulin resistance (IR) is characterized by a diminished sensitivity to insulin in peripheral tissues [7] and is a pivotal factor in the progression to diabetes. Enhancing β-cell function and insulin sensitivity can stabilize prediabetes and promote a return to normoglycemia. Although the euglycemic-hyperinsulinemic clamp (HEC) is recognized as the benchmark for assessing IR, its high cost and complexity render it impractical for widespread clinical application [8]. Consequently, there is a drive to identify efficient and cost-effective markers. The triglyceride glucose (TyG) index and the triglyceride to high-density lipoprotein cholesterol (TG/HDL-C) ratio have emerged as reliable surrogates for IR [9, 10], given their strong correlation with HEC and their suitability for large-scale epidemiologic studies and clinical practice [11, 12].

Recent studies have established a link between these two parameters and the incidence of prediabetes or diabetes among individuals in the general population [13–15]. However, the precise relationship between these markers and the risk of diabetes progression in prediabetic individuals is not fully understood. To date, only limited research has explored the relationship between the TG/HDL-C ratio and the incidence of diabetes in prediabetic individuals [16], Furthermore, whether the TyG index can accurately predict the advancement to diabetes remains to be determined.

Given the ease and affordability of measuring the TyG and TG/HDL-C ratios, understanding their relationship with disease progression, especially considering the widespread occurrence of prediabetes and its associated complications, could significantly aid in prevention and treatment strategies. This study used multicenter physical examination data from the China Fukang Medical Group to explore the predictive value of these indices for diabetes risk in the prediabetic population.

## Methods

### Study design and participants

This retrospective cohort analysis leveraged longitudinal data from the China Rich Medical Group’s multicenter health screening cohort spanning from 2010 to 2016. Eligible participants underwent at least two health screenings within this timeframe. The original cohort, comprising 685,277 Chinese adults over 20 years of age, was established to explore the role of obesity in diabetes development [17]. After applying exclusion criteria consistent with previous study aims, 211,833 participants with documented type 2 diabetes outcomes were selected for analysis. Anonymized data were made available on the Dryad digital repository for secondary analysis. No additional ethical approval was required for subsequent analyses, as the initial result was granted by the Rich Healthcare Subcommittee Review Board. This research adhered to the STROBE guidelines and the Declaration of Helsinki principles.

**Fig. 1:**
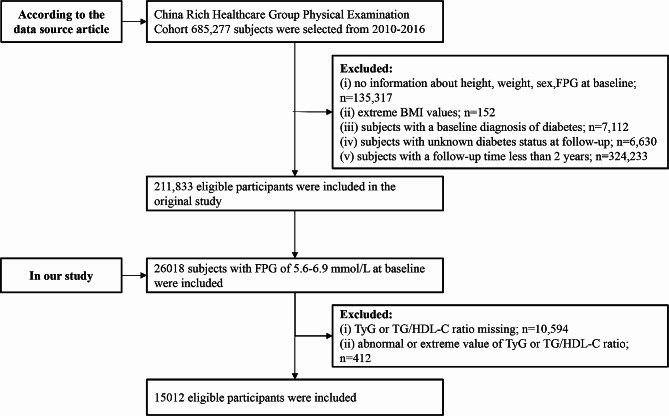
Flowchart of study participants selection

The study population comprised 26,018 baseline prediabetic individuals, defined by measurements ranging from 5.6 to 6.9 mmol/L, following the American Diabetes Association criteria. The transition from prediabetes to diabetes was the primary dependent variable, while the TyG index and TG/HDL-C ratio served as independent variables. Participants with abnormal (*n* = 412) or missing (*n* = 10,594) data for these indices were excluded, yielding a final sample size of 15,012 participants. The process of subject inclusion and exclusion is illustrated in Fig. 1.

### Baseline indicator measurement assessment

In the initial phase of the study, trained investigators systematically collected demographic data, including age and sex, as well as lifestyle factors such as alcohol consumption and smoking, and family history of diabetes using a self-administered questionnaire. Smoking status was ascertained by asking participants, “Do you smoke?” with options. Those who reported never smoking were classified as “never smokers”. Former smokers were identified as those who had smoked but since quit smoking, while current smokers were those who continued smoking. Alcohol consumption was determined by inquiring, “Have you consumed any alcoholic beverages in the past year?” Nondrinkers were defined as individuals who had not consumed alcohol, while current drinkers were those who continued to consume alcohol daily. Former drinkers were those who had ceased alcohol consumption. Precision was applied in recording anthropometric data including body weight and height, which allowed for the subsequent computation of the body mass index (BMI). Concurrently, blood pressure was measured utilizing a mercury sphygmomanometer.

Fasting blood samples were obtained after at least 10 h of fasting and processed within 2 h of collection. A Beckman 5800 automatic biochemical analyser was used to assess common biochemical markers, including TG, total cholesterol (TC), fasting plasma glucose (FPG), HDL-C, low-density lipoprotein cholesterol (LDL-C), blood urea nitrogen (BUN), creatinine (Cr), alanine aminotransferase (ALT), and aspartate aminotransferase (AST).

### Variables

The calculation of the TyG index was performed utilizing the specific formula. TyG Index = Ln ((fasting plasma glucose (mmol/L)×18) × (triglyceride (mmol/L)×88.5)/2) [11], and the TG/HDL-C ratio was determined by another formula: TG/HDL-c ratio = (triglyceride (mmol/L) ×88.5)/(high-density lipoprotein cholesterol (mmol/L) ×38.67) [18].

### Outcome measures

The primary outcome was the progression from prediabetes to diabetes, with prediabetes defined by baseline FPG levels between 5.6 and 6.9 mmol/L. Diabetes was identified through self-reports or an FPG level of concentration of 7.0 mmol/L or higher at the final visit [19].

### Statistical analysis

To mitigate bias from missing data, we utilized multiple imputations based on chained equations to estimate missing values. Continuous variables are presented as median values alongside interquartile ranges (IQRs) or means accompanied by their standard deviations (SDs). In contrast, categorical variables are expressed in terms of frequency, marked as a percentage. To evaluate the significant differences among the quartiles of the TyG index or the TG/HDL-C ratio, methods such as chi-square tests, Kruskal‒Wallis tests, and variance analysis were utilized.

Collinearity between the two indices and other covariates was diagnosed using tolerance and variance inflation factors. The associations between either the TyG index or the TG/HDL-C ratio and the risk of diabetes were gauged using the Cox proportional hazards model, with adjustments for various covariates. Prior to modelling, the assumption of proportional hazards was verified through Schoenfeld residuals. Model 1 was unadjusted, while Model 2 included adjustments for sex, age, HDL-C, LDL-C, AST, ALT, blood pressure, BMI, Cr, BUN, alcohol consumption status, smoking status,, and familial diabetes history. Linear trends were discerned through the median score for each quartile, and the impact of a standard deviation increase in the index on the outcome risk was evaluated as a continuous variable. Nonlinear correlation were explored using a fully adjusted Cox model with smooth curve fitting functions. The predictive efficacy of the two indices was assessed via the area under the curve (AUC) from receiver operator characteristic (ROC) curve analysis.

Potential heterogeneity in the relationship between the two markers and the risk of diabetes was investigated across prevalent phenotypes, including sex, diabetes family history, BMI categories [20], and age groups based on WHO criteria [21]. Interactions among groups were compared using likelihood ratio tests.

To ensure robustness, four sensitivity analyses were conducted: (1) aligning disease definitions with the WHO criteria for T2DM and impaired fasting glucose; (2) employing a competing risk model analysis for three possible outcomes (normoglycaemia, prediabetes, diabetes); (3) Conducting an analysis on the original dataset without multiple imputation; (4) Adjusting for the interaction of sex or age with the TyG index or the TG/HDL-C ratio in the Cox proportional hazards model. Statistical analyses were conducted utilizing R-4.3.0, SAS 9.4, and Empower^®^2.0. *P* < 0.05 was considered to indicate statistical significance.

## Results

### Participant characteristics

Table 1 outlines the characteristics of the study participants, divided according to TyG index quartiles, while Table 2 shows the same for the TG/HDL-C ratio quartiles. Among the 15,012 prediabetic subjects, the median values for the TyG index and TG/HDL-C ratio were 8.81 (IQR 8.41–9.21) and 2.51 (IQR 1.58–3.93), respectively. An increase in the two indices corresponded with older age, male sex, higher blood pressure, greater BMI, lower levels of TC, TG, HDL-C, and LDL-C, and diminished ALT, AST, and Cr levels. Current alcohol consumption and smoking status were also linked to the two highest baseline indices. The distributions of the two indices are depicted in Supplementary File 2: Fig. S1, which shows a normal distribution for the TyG index and a right-skewed distribution for the TG/HDL-C ratio.

**Table 1:** Baseline characteristics of the study population according to the TyG quartile groups

	TyG quartile				*P*-value
	Quartile1 (< 8.433)	Quartile 2 (8.413–8.816)	Quartile 3 (8.816–9.218)	Quartile 4 (≥ 9.218 )	
No. of subjects	3764	3741	3754	3753	
**Sociodemographic parameters**					
Age; years, medians (Q1-Q4)	46.00 (36.00–58.00)	52.00 (40.00–61.00)	52.20 (42.00–61.00)	53.00 (43.00–61.00)	< 0.001
Sex, n(%)					< 0.001
Male	1914 (50.85)	2331 (62.31)	2615 (69.66)	2809 (74.85)	
Female	1850 (49.15)	1410 (37.69)	1139 (30.34)	944 (25.15)	
**Anthropometric parameters**					
BMI; kg/m^2^, mean (SD)	23.07 ± 3.01	24.53 ± 3.16	25.45 ± 3.19	26.11 ± 3.09	< 0.001
**Clinical parameters**					
SBP; mmHg, mean (SD)	122.59 ± 17.03	126.95 ± 17.58	129.15 ± 17.48	131.03 ± 17.58	< 0.001
DBP; mmHg, mean (SD)	75.15 ± 10.86	77.66 ± 10.84	79.62 ± 11.03	81.24 ± 11.04	< 0.001
Family history of diabetes, n(%)	103 (2.74)	93 (2.49)	98 (2.61)	98 (2.61)	0.927
**Biochemical parameters**					
FPG; mmol/L, mean (SD)	5.86 ± 0.26	5.93 ± 0.30	5.97 ± 0.32	6.05 ± 0.35	< 0.001
TC; mmol/L, mean (SD)	4.62 ± 0.84	4.94 ± 0.87	5.17 ± 0.92	5.37 ± 0.94	< 0.001
TG; mmol/L, mean (SD)	0.73 ± 0.17	1.20 ± 0.14	1.72 ± 0.20	2.99 ± 0.91	< 0.001
HDL-C; mmol/L, mean (SD)	1.45 ± 0.29	1.38 ± 0.32	1.30 ± 0.27	1.24 ± 0.27	< 0.001
LDL-C, mmol/L, medians (Q1-Q4)	2.63 (2.25–3.07)	2.89 (2.48–3.33)	3.04 (2.59–3.51)	2.99 (2.54–3.50)	< 0.001
ALT, U/L, medians (Q1-Q4)	16.40 (12.30–23.00)	20.30 (15.00-29.40)	24.00 (17.40-35.08)	28.00 (20.00-40.90)	< 0.001
AST, U/L, medians (Q1-Q4)	21.72 (17.60–26.90)	24.00 (19.30-29.23)	25.00 (20.21-31.00)	27.00 (21.70–33.60)	< 0.001
BUN, mmol/L, medians (Q1-Q4)	4.85 (4.07–5.70)	4.87 (4.11–5.75)	4.92 (4.16–5.80)	4.85 (4.10–5.70)	0.129
Cr, umol/L, medians (Q1-Q4)	68.00 (57.60–80.60)	72.30 (60.70–82.90)	74.50 (62.60–84.10)	75.30 (64.90–85.20)	< 0.001
**Lifestyle parameters**					
Smoking status, n(%)					< 0.001
Current	655 (17.40)	862 (23.04)	1038 (27.65)	1175 (31.31)	
Ever	168 (4.46)	172 (4.60)	204 (5.43)	208 (5.54)	
Never	2941 (78.13)	2707 (72.36)	2512 (66.92)	2370 (63.15)	
Drinking status, n(%)					< 0.001
Current	116 (3.08)	160 (4.28)	169 (4.50)	291 (7.75)	
Ever	578 (15.36)	622 (16.63)	711 (18.94)	805 (21.45)	
Never	3070 (81.56)	2959 (79.10)	2874 (76.56)	2657 (70.80)	

Values were expressed as mean (standard deviation) or medians (quartile1- quartile 4) or n (%)

Abbreviations: SD: standard deviation; TyG: triglyceride glucose index; BMI: body mass index; SBP: systolic blood pressure; DBP: diastolic blood pressure; FPG: fasting plasma glucose; TG: triglyceride; TC: total cholesterol; HDL-C: high-density lipoprotein cholesterol; LDL-C: low-density lipoprotein cholesterol; ALT: alanine aminotransferase; AST: aspartate aminotransferase; BUN: blood urea nitrogen; Cr: creatinine.

**Table 2:** Baseline characteristics of the study population according to the TG/HDL-C ratio quartile groups

	TG/HDL-C ratio quartile				*P*-value
	Q1 (< 1.586)	Q2 (1.586–2.504)	Q3 (2.504–3.938)	Q4 (≥ 3.938 )	
No. of subjects	3778	3780	3772	3777	
**Sociodemographic parameters**					
Age; years, medians (Q1-Q4)	48.00 (37.00–59.00)	51.00 (39.00–61.00)	52.00 (42.00–61.00)	52.00 (42.00–61.00)	< 0.001
Sex, n(%)					< 0.001
Male	1790 (47.38)	2366 (62.59)	2668 (70.73)	42,921 (77.34)	
Female	1988 (52.62)	1414 (37.41)	1104 (29.27)	856 (22.66)	
**Anthropometric parameters**					
BMI; kg/m^2^, mean (SD)	22.96 ± 3.04	24.59 ± 3.13	25.46 ± 3.12	26.17 ± 3.07	< 0.001
**Biochemical parameters**					
SBP; mmHg, mean (SD)	123.11 ± 17.57	127.38 ± 17.65	129.11 ± 17.37	130.12 ± 17.36	< 0.001
DBP; mmHg, mean (SD)	75.23 ± 10.76	77.94 ± 11.11	79.64 ± 11.12	80.92 ± 10.90	< 0.001
FPG; mmol/L, mean (SD)	5.89 ± 0.29	5.92 ± 0.30	5.97 ± 0.32	6.00 ± 0.33	< 0.001
TC; mmol/L, mean (SD)	4.78 ± 0.88	4.98 ± 0.91	5.10 ± 0.93	5.24 ± 0.94	< 0.001
TG; mmol/L, mean (SD)	0.75 ± 0.21	1.22 ± 0.23	1.74 ± 0.35	2.98 ± 1.05	< 0.001
HDL-C; mmol/L, medians (Q1-Q4)	1.53 (1.36–1.71)	1.38 (1.23–1.54)	1.27 (1.12–1.43)	1.10 (0.97–1.28)	< 0.001
LDL-C; mmol/L, medians (Q1-Q4)	2.72 (2.31–3.16)	2.91 (2.48–3.37)	2.98 (2.54–3.45)	2.93 (2.48–3.45)	< 0.001
ALT; U/L, medians (Q1-Q4)	16.30 (12.20–23.00)	20.80 (15.00-29.25)	24.00 (17.10–35.00)	28.50 (20.00-41.60)	< 0.001
AST; U/L, medians (Q1-Q4)	21.90 (18.00–26.00)	23.40 (19.70–28.30)	24.00 (20.50–29.00)	26.00 (21.50–32.00)	< 0.001
BUN; mmol/L, medians (Q1-Q4)	4.86 (4.09–5.72)	4.90 (4.14–5.80)	4.90 (4.14–5.77)	4.84 (4.10–5.70)	0.043
Cr; umol/L, medians (Q1-Q4)	67.00 (57.00-79.40)	72.80 (61.00–83.00)	75.00 (63.65-85.00)	75.60 (65.10–85.10)	< 0.001
Family history of diabetes, n(%)	101 (2.67)	88 (2.33)	103 (2.73)	103 (2.73)	0.646
**Lifestyle parameters**					
Smoking status, n(%)					< 0.001
Current	683 (18.08)	843 (22.3)	950 (25.19)	1202 (31.83)	
Ever	160 (4.24)	167 (4.43)	203 (5.38)	259 (6.85)	
Never	2935 (77.68)	2770 (73.27)	2619 (69.43)	2316 (61.32)	
Drinking status, n(%)					< 0.001
Current	146 (3.87)	163 (4.32)	205 (5.43)	233 (6.17)	
Ever	578 (15.31)	620 (16.4)	700 (18.55)	832 (22.04)	
Never	3053 (80.82)	2997 (79.28)	2867 (76.02)	2712 (71.79)	

Values were expressed as mean (standard deviation) or medians (quartile1- quartile 4) or n (%)

Abbreviations: BMI: body mass index; SBP: systolic blood pressure; DBP: diastolic blood pressure; FPG: fasting plasma glucose; TG: triglyceride; TC: total cholesterol; HDL-C: high-density lipoprotein cholesterol; LDL-C: low-density lipoprotein cholesterol; ALT: alanine aminotransferase; AST: aspartate aminotransferase; BUN: blood urea nitrogen; Cr: creatinine

### Association of baseline TyG or TG/HDL-C ratio with diabetes

Schoenfeld residual plots for the two indices over time(Supplementary File 2: Fig. S2) confirmed the validity of the proportional hazards assumption for the Cox proportional hazards model. After testing for collinearity, LDL-C, TG, and TC were omitted from further multivariable models due to a variance inflation factor above the threshold of 5, suggesting multicollinearity (Supplementary File 1: Table S1).

**Fig. 2:**
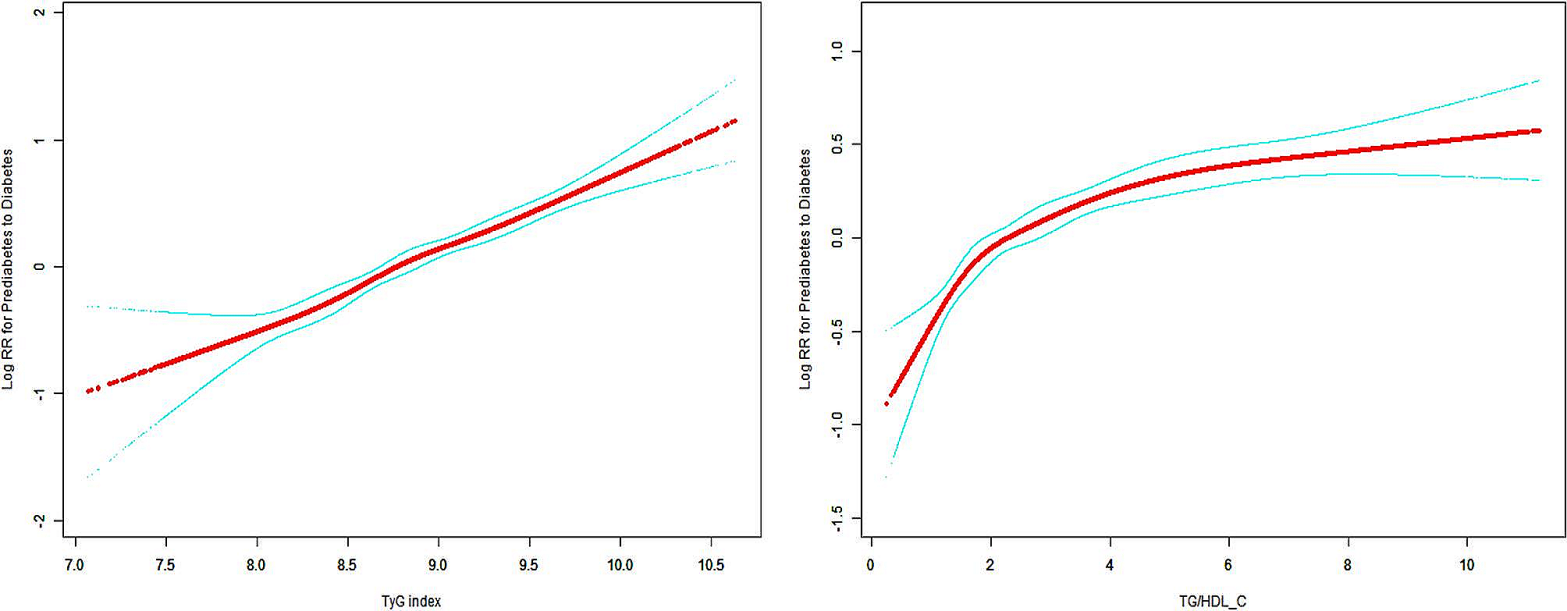
Dose-response association of the TyG index or TG/HDL-c ratio with the risk of diabetes in participants with prediabetes. The solid line represents the nonlinear dose-response, and the dotted lines represent the 95% confidence interval

Over a median follow-up duration of 2.87 years (IQR 2.08–3.57; totaling 34,268 person-years), 1,730 (11.5%) prediabetes patients developed type 2 diabetes. The risk of diabetes increased with increasing TyG index values, even after adjusting for sociodemographic variables. The adjusted risk ratios for diabetes in relation to the lowest quartile for both indices showed a progressive increase from quartile Q2 to Q4 for TyG (1.52, 95% CI: 1.28–1.81 to 2.59, 95% CI: 2.20–3.05) and for the TG/HDL-C ratio (1.31, 95% CI: 1.11–1.55 to 2.03, 95% CI: 1.71–2.40). This indicates a significant association between higher quartiles of these indices and the risk of diabetes. Significant trends were observed across quartiles for both indices (*P* < 0.001). Notably, for every one SD increase in the logarithmically transformed TyG index, the likelihood of developing diabetes increased by 23%. Similarly, the risk increased by 43% for each increase in the TG/HDL-C ratio. (Table 3). Multivariate adjusted analysis using limited cubic spline revealed a linear correlation between the progression to diabetes and the two indices in question (TyG index: *p* = 0.031; TG/HDL-C index: *P* < 0.001) (Fig. 2).

**Table 3:** Multivariable Cox regression analysis of the association between the TyG index and TG/HDL-C ratio with the risk of diabetes in patients with prediabetes

	No. of case	Person-years	Incidence density (100 person-year)	HR (95%CI)	
				Model I	Model II
TyG				2.11 (1.94, 2.30)	1.86 (1.70, 2.04)
TyG (quartile)					
Q1	213	11014.94	1.93	Ref	Ref
Q2	369	11014.92	3.35	1.76 (1.48, 2.08)	1.52 (1.28, 1.81)
Q3	469	11076.74	4.23	2.21 (1.88, 2.59)	1.82 (1.54, 2.15)
Q4	679	11183.52	6.07	2.35 (2.04, 2.93)	2.59 (2.20, 3.05)
P-trend				< 0.001	< 0.001
Per 1 SD increase in TyG				1.52 (1.44, 1.59)	1.43 (1.36, 1.51)
TG/HDL-C ratio				1.09 (1.07, 1.11)	1.10 (1.07, 1.12)
TG/HDL-C ratio (quartile)					
Q1	259	10742.76	2.41	Ref	Ref
Q2	370	10957.70	3.38	1.31 (1.12, 1.54)	1.31 (1.11, 1.55)
Q3	486	11111.29	4.37	1.69 (1.46, 1.97)	1.59 (1.35, 1.87)
Q4	615	11455.92	5.37	1.97 (1.70, 2.28)	2.03 (1.71, 2.40)
P-trend				< 0.001	< 0.001
Per 1 SD increase in TG/HDL-C ratio				1.20 (1.15, 1.24)	1.23 (1.17, 1.28)

Abbreviations: HR: hazard ratios; SD: standard deviation; CI: confidence interval; other abbreviations as in Table 1

Model I : we did not adjust other covariates

Model II: Adjusted for age, sex, SBP, DBP, HDL-C, ALT, AST, BUN, Cr, BMI, family history of diabetes, smoking status, drinking status

### **Ability of both indices for assessing diabetes risk**

**Fig. 3:**
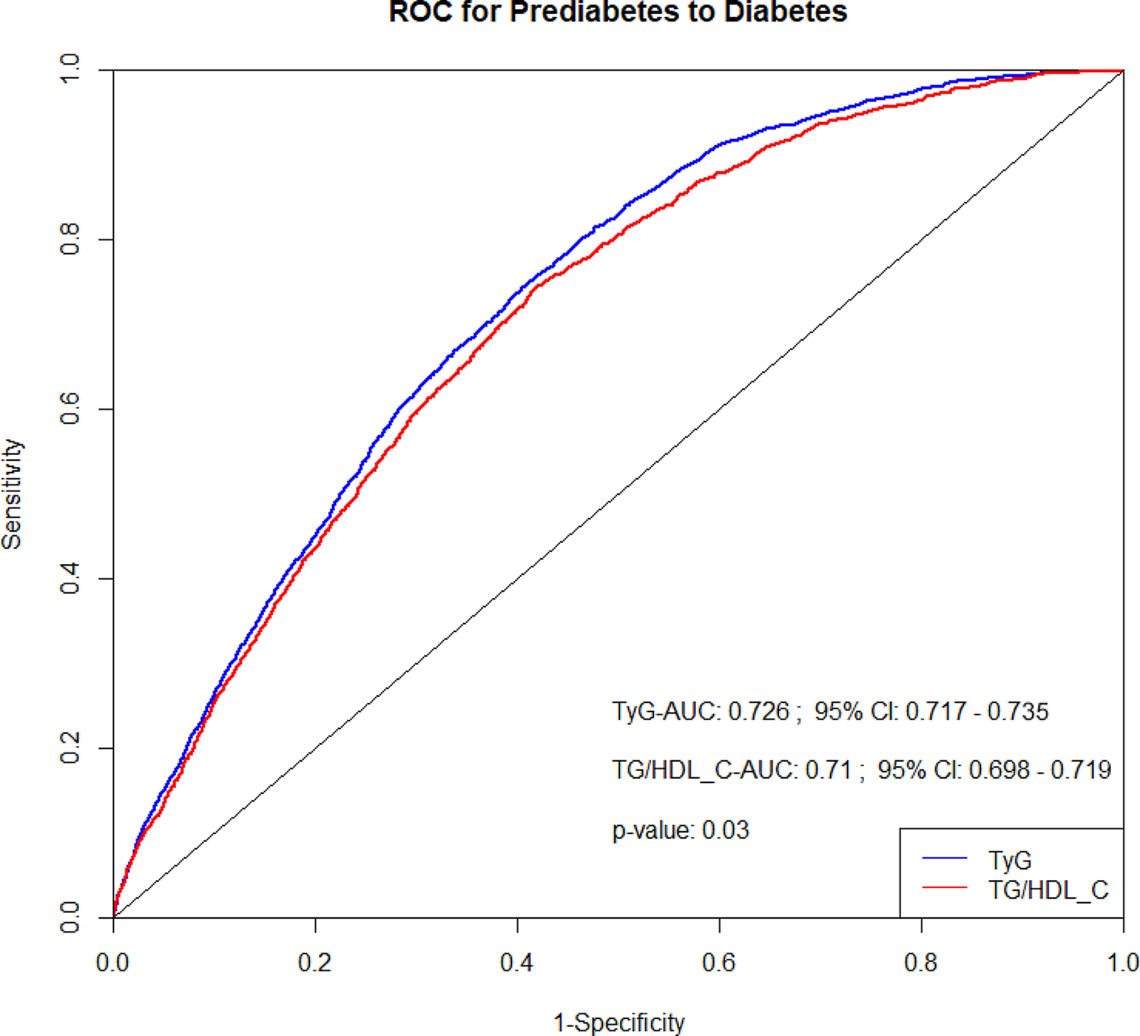
Receiver operating characteristic curves for the TyG index or TG/HDL-c ratio in predicting incident diabetes. Adjusted for age, sex, SBP, DBP, HDL-C, ALT, AST, BUN, Cr, BMI, family history of diabetes, smoking status, and drinking status. ROC: Receiver operating characteristic; AUC: area under the curve

In the diabetes risk prediction model, the AUCs for the TyG index and the TG/HDL-C ratio were 0.726 (95% CI: 0.717–0.735) and 0.710 (95% CI: 0.698–0.719), respectively, with a notable difference between them (*P* = 0.03) (Fig. 3). The positive and negative likelihood ratios for the TyG index were 1.756 and 0.323, respectively, and for the TG/HDL-C ratio, they were 1.824 and 0.442, respectively (Supplementary File 1: Table S2).

In the diabetes risk prediction model, the AUCs for the TyG index and the TG/HDL-C ratio were 0.726 (95% CI: 0.717–0.735) and 0.710 (95% CI: 0.698–0.719), respectively, with significant differences between them (*P* = 0.03) (Fig. 3). The positive and negative likelihood ratios for the TyG index were 1.756 and 0.323, respectively. Similarly, the TG/HDL-C ratios were 1.824 and 0.442, respectively (Supplementary File 1: Table S2).

### Subgroup analysis and sensitivity analysis

Stratification variables included age, sex, BMI, and family history of diabetes. The relationship between glycemic deterioration and the TyG index was found to be influenced by age and sex (*P* for interaction < 0.05). Subgroup analyses by family history and BMI, revealed no significant interaction between the TyG index and the risk of diabetes (*P* > 0.05). Notably, the TyG index was not associated with an increased risk of diabetes among participants with a family history of the disease (HR:1.33, 95% CI: 0.84–2.12). The TG/HDL-C ratio exhibited analogous results. (Table 4). Competing risk model analysis, the original dataset analysis without multiple imputation, adjusting for the interaction of sex or age with the two indices, and sensitivity analyses using the 1999 WHO criteria definition confirmed the stability of the relationship between the two indices and glycemic deterioration (Supplementary File 1: Table S3), demonstrating a significant correlation (*P* < 0.001).

**Table 4:** Stratified analyses of the associations between the TyG index or TG/HDL-C ratio and the risk of diabetes in patients with prediabetes

	HR (95%CI) in TyG and the risk of diabetes	HR (95%CI) in TG/HDL-C ratio and the risk of diabetes
**Sex**		
Male	1.78 (1.60, 1.99)	1.09 (1.06, 1.12)
Female	2.19 (1.82, 2.63)	1.17 (1.12, 1.22)
*P*-interaction	0.005	0.004
**Age**,** years**		
<45	3.32 (2.67, 4.13)	1.21 (1.15, 1.27)
≥ 45	1.76 (1.59, 1.94)	1.09 (1.06, 1.11)
*P*-interaction	< 0.001	< 0.001
**BMI**,** kg/m**^**2**^		
<25	2.07 (1.80, 2.39)	1.13 (1.09, 1.17)
≥ 25	1.88 (1.67, 2.12)	1.09 (1.07, 1.12)
*P*-interaction	0.305	0.122
**Family history of diabetes**		
No	1.99 (1.81, 2.19)	1.11 (1.08, 1.13)
Yes	1.33 (0.84, 2.12)	1.06 (0.94, 1.19)
*P*-interaction	0.096	0.441

Abbreviations: HR: hazard ratios; CI: confidence interval; other abbreviations as in Table 1

Note: Models adjusted for the same covariates as in model II (Table 3), except for the stratification variable.

## Discussion

In this longitudinal, multicenter health examination cohort of adult prediabetes patients, high initial TyG index and TG/HDL-C ratio were identified as significant predictors of diabetes development. The correlation remained robust after accounting for variables that could confound the results. When comparing the highest quartile to the lowest quartile, there was a significant increase in the risk of diabetes, with a 2.03-fold increase in the TyG index and a 2.59-fold increase in the TG/HDL-C ratio greater risk. Moreover, by employing smooth curve fitting techniques, a linear correlation was observed between both indices and diabetes risk, with the TyG index demonstrating superior predictive capabilities. Sensitivity analysis revealed consistent results, and subgroup analyses indicated that age and sex were influential modifiers of the disease.

Previous studies have demonstrated that individuals with prediabetes have an increased likelihood of developing diabetes [22, 23], findings consistent with our findings. Over a median follow-up period of 2.87 years, we observed that 11.5% of the prediabetic patients developed diabetes. Diabetes primarily develops as a result of IR [24]. Research has shown a correlation between the risk of diabetes and both indices, which are frequently used as proxy indicators of IR [25–28]. Furthermore, elevated levels of the two indices have also been correlated with increased prediabetes risk [13, 29]. However, there is a dearth of research exploring the relationship between these indices and the risk of diabetes progression, especially among prediabetic individuals [16, 30, 31]. After adjusting for potential confounding factors, previous works revealed a positive correlation between the two parameters and diabetes risk. The current findings agree with those of earlier investigations; the analysis based on smooth curve fitting revealed an approximately linear correlation between the two parameters and progression to diabetes. Additionally, the present work demonstrated a positive correlation between the TyG index and progression to diabetes. Moreover, sensitivity analysis demonstrated the reliability of the two indices in predicting the risk of diabetes progression. These findings strengthen the body of research on the two markers of prediabetic glucose state transition risk and highlight their value as practical early indicators of subclinical disease progression.

The current analysis revealed that the TyG index exhibits a superior ability to predict diabetes risk compared to TG/HDL-C, indicating that the TyG index may be a more useful predictor of T2DM, but the underlying mechanism remains unresolved. Higher TyG index values are linked to a greater likelihood of diabetes development in prediabetic patients, which may be attributed to IR [11, 32]. In accordance with Romero et al.‘s study [11], we hypothesized that the connection between the two parameters and diabetes risk is mediated by IR. Since IR can lead to aberrant blood glucose levels, this may explain why the two indices have a robust predictive capacity for prediabetes. Additionally, pancreatic beta-cell dysfunction could account for the association between these two indices and disease progression [33, 34]. Beta cells are susceptible to glucotoxicity and lipotoxicity, and it is well established that elevated glucose levels increase reactive oxygen species that inflict cellular damage [35]. Furthermore, increased triglyceride levels raise ceramide and nitric oxide levels, which in turn cause beta-cell death and IR in response to glucose. Moreover, low HDL-C levels inhibit cholesterol efflux, leading to its accumulation in beta cells, causing beta-cell malfunction, elevated blood sugar, reduced insulin production, and eventual beta-cell loss [36]. Collectively, these mechanisms may underlie the correlation between the two parameters and the risk of diabetes [37].

### Strengths and limitations

The strengths of this study lie in its detailed examination of the nonlinear relationship between the two indices and diabetes risk among the prediabetic population, its use of ROC analysis to evaluate the predictive ability, and its sensitivity analyses that consider various methodological approaches. Additionally, it includes a subgroup analysis to account for potential confounding factors and employs established quality control methods for measuring study variables. However, this work has several limitations. First, the findings are based on FPG due to database limitations; the absence of information on oral glucose tolerance tests and glycated haemoglobin may lead to an underestimation of the incidence of diabetes. However, since nondiabetic individuals seldom undergo oral glucose tolerance testing, a diagnosis based on FPG may be sufficiently accurate for representing affected individuals. Second, the relatively short median follow-up period that may not adequately reflect the risk relationship. Third, the study population was limited, and further research is required to enhance the identification and prognosis of diabetes development in other countries and ethnic groups. Fourth, residual confounding due to unavailable data on diet, physical activity, certain medical conditions, or metabolic parameters may introduce unobserved variables even after comprehensive adjustment. Fifth, the present study did not include nonalcoholic fatty liver disease (NAFLD), which is prevalent among prediabetic patients and could influence the findings. Finally, the study evaluated only the baseline indices, and tracking long-term changes could provide additional insights.

## Conclusion

This study underscores the association between higher initial TyG or TG/HDL-C levels and heightened T2DM risk in prediabetic individuals. The TyG index, in particular, shows superior predictive power for diabetes risk, suggesting that efforts to prevent the onset of diabetes may benefit from targeting the reduction in TyG levels in patients with prediabetes.

### Electronic supplementary material

Below is the link to the electronic supplementary material.

Supplementary Material 1. Table S1: Diagnostic for collinearity between the TyG index or TG/HDL-C ratio and other covariates when the risk of diabetes is the dependent variable. Table S2: Areas under the receiver operating characteristic curves for the TyG index and TG/HDL-C ratio in identifying diabetes. Table S3: Sensitivity analysis.

Supplementary Material 2. Fig S1: Histograms showing the population distribution of the TyG index and TG/HDL_C ratio.

Supplementary Material 3. Fig S2: Schoenfeld residual plot. (A) Schoenfeld residual plot of the TyG index changes over time with prediabetes to diabetes as the dependent variable; (B) Schoenfeld residual plot of the TG/HDL_C ratio changes over time with prediabetes to diabetes as the dependent variable. The *P*-value of Schoenfeld Residuals test result is larger than 0.05, indicating that the TyG index and TG/HDL_C ratio are not time dependent variables and can be analyzed by the Cox Proportional Hazards Model.

Supplementary Material 4

Supplementary Material 5

Supplementary Material 6

Supplementary Material 7

## Data Availability

The raw data can be downloaded from the ‘DATADRYAD’ database (www. Datadryad.org). Dryad Digital Rep. https://datadryad.org/stash/dataset/doi:10.5061/dryad.ft8750v.
