# Supplementary material for: A longitudinal study on the impact of the TyG Index and TG/HDL-C ratio on the risk of type 2 diabetes in Chinese patients with prediabetes
Source: Lipids Health Dis. 2024 Aug 22;23:262. doi: 10.1186/s12944-024-02239-1 (PMC11340070; doi:10.1186/s12944-024-02239-1)
Supplement: Supplementary file 1 — Supplementary Material 1. Table S1: Diagnostic for collinearity between the TyG index or TG/HDL-C ratio and other covariates when the risk of diabetes is the dependent variable. Table S2: Areas under the receiver operating characteristic curves for the TyG index and TG/HDL-C ratio in identifying diabetes. Table S3: Sensitivity analysis. [file 12944_2024_2239_MOESM1_ESM.docx]

**Supporting information**

**Table S1**: Diagnostic for collinearity between the TyG index or TG/HDL-C ratio and other covariates when the risk of diabetes is the dependent variable.

|  | **Tolerance** | **VIF** |  |  | **Tolerance** | **VIF** |
| --- | --- | --- | --- | --- | --- | --- |
| TyG | 0.10 | 8.81 |  | TG/HDL-C | 0.06 | 15.93 |
| Age | 0.72 | 1.37 |  | Age | 0.75 | 1.32 |
| Sex | 0.53 | 1.87 |  | Sex | 0.53 | 1.86 |
| BMI | 0.74 | 1.33 |  | BMI | 0.76 | 1.30 |
| SBP | 0.52 | 1.92 |  | SBP | 0.52 | 1.92 |
| DBP | 0.55 | 1.79 |  | DBP | 0.55 | 1.79 |
| TC | 0.14 | 6.93 |  | TC | 0.14 | 6.93 |
| TG | 0.12 | 8.07 |  | TG | 0.07 | 12.95 |
| HDL-C | 0.68 | 1.45 |  | HDL-C | 0.32 | 3.07 |
| LDL-C | 0.16 | 5.89 |  | LDL-C | 0.16 | 5.89 |
| ALT | 0.27 | 3.58 |  | ALT | 0.27 | 3.57 |
| AST | 0.30 | 3.25 |  | AST | 0.30 | 3.25 |
| BUN | 0.86 | 1.15 |  | BUN | 0.86 | 1.15 |
| Cr | 0.54 | 1.84 |  | Cr | 0.54 | 1.84 |
| Family history of diabetes | 0.99 | 1.03 |  | Family history of diabetes | 0.99 | 1.03 |
| Smoking status | 0.91 | 1.08 |  | Smoking status | 0.91 | 1.08 |
| Drinking status | 0.92 | 1.09 |  | Drinking status | 0.91 | 1.09 |

VIF: variance inflation factor; VIF = 1/(1-R^2^). Abbreviations as in Table 1.

Note: The variables with VIF>5 will be regarded as collinear variables and cannot be included in the multiple regression model.

**Table S2:** Areas under the receiver operating characteristic curves for the TyG index and TG/HDL-C ratio in identifying diabetes.

|  | **AUC** | **95% CI** | **Sensitivity** | **Specificity** | **Youden's index** | **Positive likelihood ratio** | **Negative likelihood ratio** |
| --- | --- | --- | --- | --- | --- | --- | --- |
| TyG | 0.726 | (0.717, 0.735) | 0.829 | 0.527 | 0.357 | 1.756 | 0.323 |
| TG/HDL-C ratio | 0.710 | (0.698, 0.719) | 0.735 | 0.596 | 0.332 | 1.824 | 0.442 |

Abbreviations: AUC Area under the curve, ROC Receiver Operator Characteristic curve, TyG index triglyceride glucose index, TG triglyceride, HDL-C high-density lipoprotein cholesterol.

**Table S3:** Sensitivity analysis.

|  | **Sensitivity-1 (HR,95%CI, P)** | **Sensitivity-2 (HR,95%CI, P)** | **Sensitivity-3 (HR,95%CI, P)** | **Sensitivity-4**  **(HR,95%CI, P)** |
| --- | --- | --- | --- | --- |
| TyG | 1.25 (1.13, 1.38), <0.001 | 2.00 (1.49, 2.69), <0.001 | 1.86 (1.70, 2.04), <0.001 | 3.74 (2.34, 5.97), <0.001 |
| TyG (quartile) |  |  |  |  |
| Q1 | Ref. | Ref. | Ref.. | Ref. |
| Q2 | 1.24 (1.04, 1.48), 0.015 | 1.78 (0.87,3.64), 0.112 | 1.52 (1.28, 1.81), 0.032 | 2.33 ( 0.91, 6.00), 0.077 |
| Q3 | 1.32 (1.10, 1.59), 0.002 | 3.23 (1.61, 6.48), <0.001 | 1.82 (1.54, 2.15), 0.001 | 4.53 (1.84, 11.16), <0.001 |
| Q4 | 1.51 (1.26, 1.59), <0.001 | 3.94 (1.98, 7.82), <0.001 | 2.59 (2.20, 3.05), <0.001 | 6.33 (2.67, 14.98), <0.001 |
| TG/HDL-C ratio | 1.01 (0.99, 1.03), 0.196 | 1.08 (1.05, 1.11), <0.001 | 1.10 (1.07, 1.12), <0.001 | 1.20 ( 1.08, 1.33), <0.001 |
| TG/HDL-C ratio (quartile) |  |  |  |  |
| Q1 | Ref. | Ref. | Ref.. | Ref. |
| Q2 | 1.13 (0.94, 1.35), 0.178 | 1.18 (0.98, 1.41), 0.071 | 1.31 (1.11, 1.55), 0.021 | 1.39 (0.58, 3.34), 0.451 |
| Q3 | 1.36 (1.13, 1.64), <0.001 | 1.31 (1.08, 1.57), <0.001 | 1.59 (1.35, 1.87), <0.001 | 2.44 (1.06, 5.65), 0.035 |
| Q3 | 1.34 (1.11, 1.62), 0.002 | 1.68 (1.38, 2.03), <0.001 | 2.03 (1.71, 2.40), <0.001 | 3.78 (1.69, 8.46), <0.001 |

**Sensitivity-1** was sensitivity analysis results of including 4227 subjects according to WHO's diagnostic criteria for DM and IFG of 1999. We adjusted age, sex, SBP, DBP, HDL-C, LDL-C, ALT, AST, BUN, Cr, BMI, family history of diabetes, smoking status, drinking status.

**Sensitivity-2** was sensitivity analysis results based on the results of Competing Risks Model Analysis (N=15,012) (Y: Prediabetes, Normoglycemia, Diabetes). We adjusted age, sex, SBP, DBP, HDL-C, LDL-C, ALT, AST, BUN, Cr, BMI, family history of diabetes, smoking status, drinking status.

**Sensitivity-3** was sensitivity analysis results based on the results of the original dataset without multiple imputation analysis (N=15,012). We adjusted age, sex, SBP, DBP, HDL-C, LDL-C, ALT, AST, BUN, Cr, BMI, family history of diabetes, smoking status, drinking status.

**Sensitivity-4** was sensitivity analysis results based on the results of the multiple imputation dataset (N=15,012). We further adjusted the interaction of sex* TyG and age * TyG , or of sex * TG/HDL-C ratio and age * TG/HDL-C ratio.

HR, hazard ratios; CI, confidence interval, Ref: reference; other abbreviations as in Table 1.
