# Supplementary figures and images for: A longitudinal study on the impact of the TyG Index and TG/HDL-C ratio on the risk of type 2 diabetes in Chinese patients with prediabetes
Source: Lipids Health Dis. 2024 Aug 22;23:262. doi: 10.1186/s12944-024-02239-1 (PMC11340070; doi:10.1186/s12944-024-02239-1)

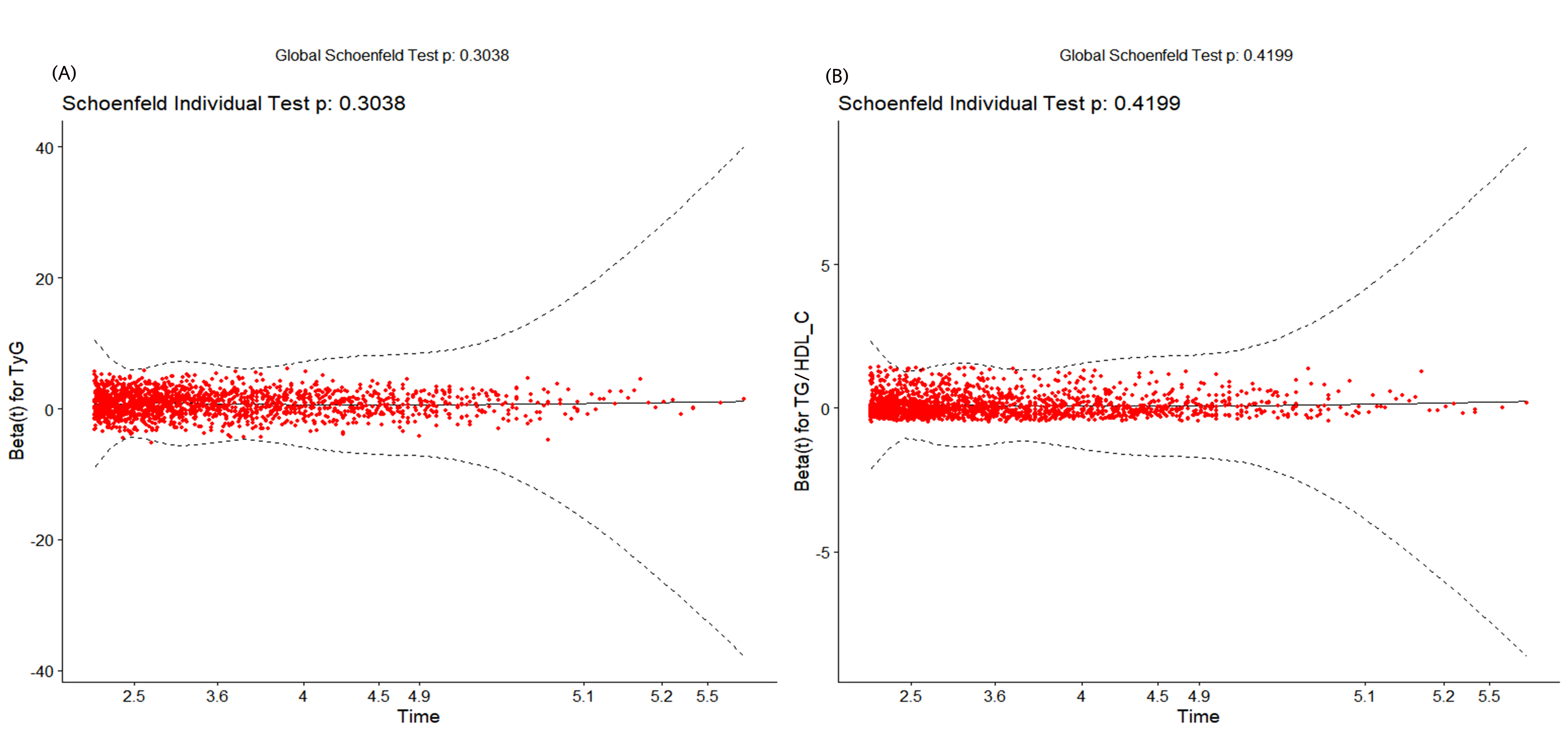

Supplement: Supplementary file 3 — Supplementary Material 3. Fig S2: Schoenfeld residual plot. (A) Schoenfeld residual plot of the TyG index changes over time with prediabetes to diabetes as the dependent variable; (B) Schoenfeld residual plot of the TG/HDL_C ratio changes over time with prediabetes to diabetes as the dependent variable. The P-value of Schoenfeld Residuals test result is larger than 0.05, indicating that the TyG index and TG/HDL_C ratio are not time dependent variables and can be analyzed by the Cox Proportional Hazards Model. [file 12944_2024_2239_MOESM3_ESM.tif]
