# Supplementary material for: A longitudinal study on the impact of the TyG Index and TG/HDL-C ratio on the risk of type 2 diabetes in Chinese patients with prediabetes
Source: Lipids Health Dis. 2024 Aug 22;23:262. doi: 10.1186/s12944-024-02239-1 (PMC11340070; doi:10.1186/s12944-024-02239-1)
Supplement: Supplementary file 4 — Supplementary Material 4 [file 12944_2024_2239_MOESM4_ESM.pdf]

Editorial Certificate

This document certifies that the manuscript has been edited to the standards by MedPeer and is now deemed ready for publication.

Document Title

A Longitudinal Study on the Impact of the Triglyceride-Glucose Index and Triglyceride to High-Density Lipoprotein Cholesterol Ratio on Progression of Prediabetes in a Chinese Population

Author	*Date Issued*
Bo Chen	June 14, 2024
