# Supplementary material for: A longitudinal study on the impact of the TyG Index and TG/HDL-C ratio on the risk of type 2 diabetes in Chinese patients with prediabetes
Source: Lipids Health Dis. 2024 Aug 22;23:262. doi: 10.1186/s12944-024-02239-1 (PMC11340070; doi:10.1186/s12944-024-02239-1)
Supplement: Supplementary file 6 — Supplementary Material 6 [file 12944_2024_2239_MOESM6_ESM.pdf]

oSR83p7TSG

By 000000 00000

WORD COUNT 2776

TIME SUBMITTED 15-JUN-2024 04:19PM

PAPER ID 109657994

INTRODUCTION

Prediabetes is a critical health state in which blood glucose levels are higher than typical but do not reach the thresholds required for type 2 diabetes mellitus (T2DM) [1]. The prevalence of prediabetes is increasing globally, with projections estimating it will affect one billion individuals by 2045 [2, 3]. Each year, 5-10% of those with prediabetes progress to T2DM. Prediabetes is linked to an increased risk of macrovascular and microvascular complications, such as stroke, peripheral arterial disease, myocardial infarction and retinopathy, neuropathy and nephropathy [3-5]. Additionally, prediabetes is linked to an elevated risk of all-cause and cardiovascular mortality [6]. Despite these risks, the majority of individuals with prediabetes remain unaware of their metabolic condition, highlighting the critical need for early detection and intervention of modifiable factors.

Insulin resistance (IR) is characterized by a diminished sensitivity to insulin in peripheral tissues [7] and is a pivotal factor in the progression to diabetes. Enhancing β-cell function and insulin sensitivity can stabilize prediabetes and promote a return to normoglycemia. Although the euglycemic-hyperinsulinemic clamp (HEC) is recognized as the benchmark for assessing IR, its high cost and complexity render it impractical for widespread clinical application [8]. Consequently, there is a drive to identify efficient and cost-effective markers. The triglyceride glucose (TyG) index and the triglyceride to high-density lipoprotein cholesterol (TG/HDL-C) ratio have emerged as reliable surrogates for IR [9, 10], given their strong correlation with HEC and their suitability for large-scale epidemiologic studies and clinical practice [11, 12].

Recent studies have established a link between these two parameters and the incidence of prediabetes or diabetes among individuals in the general population [13-15]. However, the precise relationship between these markers and the risk of diabetes progression in prediabetic individuals is not fully understood. To date, only limited research has explored the relationship between the TG/HDL-C ratio and the incidence of diabetes in prediabetic individuals [16], Furthermore, whether the TyG index can accurately predict the advancement to diabetes remains to be determined.

Given the ease and affordability of measuring the TyG and TG/HDL-C ratios, understanding their relationship with disease progression, especially considering the widespread occurrence of prediabetes and its associated complications, could significantly aid in prevention and treatment strategies. This study used multicenter physical examination data from the China Fukang Medical Group to explore the predictive value of these indices for diabetes risk in the prediabetic population.

METHODS

Study Design and Participants

This retrospective cohort analysis leveraged longitudinal data from the China Rich

Medical Group's multicenter health screening cohort spanning from 2010 to 2016. Eligible participants underwent at least two health screenings within this timeframe. The original cohort, comprising 685,277 Chinese adults over 20 years of age, was established to explore the role of obesity in diabetes development [17]. After applying exclusion criteria consistent with previous study aims, 211,833 participants with documented type 2 diabetes outcomes were selected for analysis. Anonymized data were made available on the Dryad digital repository for secondary analysis. No additional ethical approval was required for subsequent analyses, as the initial result was granted by the Rich Healthcare Subcommittee Review Board. This research adhered to the STROBE guidelines and the Declaration of Helsinki principles.

The study population comprised 26,018 baseline prediabetic individuals, defined by measurements ranging from 5.6 to 6.9 mmol/L, following the American Diabetes Association criteria. The transition from prediabetes to diabetes was the primary dependent variable, while the TyG index and TG/HDL-C ratio served as independent variables. Participants with abnormal (n=412) or missing (n=10,594) data for these indices were excluded, yielding a final sample size of 15,012 participants. The process of subject inclusion and exclusion is illustrated in **Figure 1**.

Baseline Indicator Measurement Assessment

In the initial phase of the study, trained investigators systematically collected demographic data, including age and sex, as well as lifestyle factors such as alcohol consumption and smoking, and family history of diabetes using a self-administered questionnaire. Smoking status was ascertained by asking participants, "Do you smoke?" with options. Those who reported never smoking were classified as "never smokers". Former smokers were identified as those who had smoked but since quit smoking, while current smokers were those who continued smoking. Alcohol consumption was determined by inquiring, "Have you consumed any alcoholic beverages in the past year?" Nondrinkers were defined as individuals who had not consumed alcohol, while current drinkers were those who continued to consume alcohol daily. Former drinkers were those who had ceased alcohol consumption. Precision was applied in recording anthropometric data including body weight and height, which allowed for the subsequent computation of the body mass index (BMI). Concurrently, blood pressure was measured utilizing a mercury sphygmomanometer.

Fasting blood samples were obtained after at least 10 hours of fasting and processed within 2 hours of collection. A Beckman 5800 automatic biochemical analyser was used to assess common biochemical markers, including TG, total cholesterol (TC), fasting plasma glucose (FPG), HDL-C, low-density lipoprotein cholesterol (LDL-C), blood urea nitrogen (BUN), creatinine (Cr), alanine aminotransferase (ALT), and aspartate aminotransferase (AST).

Variables

The calculation of the TyG index was performed utilizing the specific formula. TyG Index = Ln ((fasting plasma glucose (mmol/L)×18) × (triglyceride (mmol/L)× 88.5)/2) [11], and the TG/HDL-C ratio was determined by another formula: TG/HDL-c ratio = (triglyceride (mmol/L) × 88.5)/(high-density lipoprotein cholesterol (mmol/L) ×38.67) [18].

Outcome Measures

The primary outcome was the progression from prediabetes to diabetes, with prediabetes defined by baseline FPG levels between 5.6 and 6.9 mmol/L. Diabetes was identified through self-reports or an FPG level of concentration of 7.0 mmol/L or higher at the final visit [19].

Statistical analysis

To mitigate bias from missing data, we utilized multiple imputations based on chained equations to estimate missing values. Continuous variables are presented as median values alongside interquartile ranges (IQRs) or means accompanied by their standard deviations (SDs). In contrast, categorical variables are expressed in terms of frequency, marked as a percentage. To evaluate the significant differences among the quartiles of the TyG index or the TG/HDL-C ratio, methods such as chi-square tests, Kruskal–Wallis tests, and variance analysis were utilized.

Collinearity between the two indices and other covariates was diagnosed using tolerance and variance inflation factors. The associations between either the TyG index or the TG/HDL-C ratio and the risk of diabetes were gauged using the Cox proportional hazards model, with adjustments for various covariates. Prior to modelling, the assumption of proportional hazards was verified through Schoenfeld residuals. Model 1 was unadjusted, while Model 2 included adjustments for sex, age, HDL-C, LDL-C, AST, ALT, blood pressure, BMI, Cr, BUN, alcohol consumption status, smoking status,, and familial diabetes history. Linear trends were discerned through the median score for each quartile, and the impact of a standard deviation increase in the index on the outcome risk was evaluated as a continuous variable. Nonlinear correlation were explored using a fully adjusted Cox model with smooth curve fitting functions. The predictive efficacy of the two indices was assessed via the area under the curve (AUC) from receiver operator characteristic (ROC) curve analysis.

Potential heterogeneity in the relationship between the two markers and the risk of diabetes was investigated across prevalent phenotypes, including sex, diabetes family history, BMI categories [20], and age groups based on WHO criteria [21]. Interactions among groups were compared using likelihood ratio tests.

To ensure robustness, two sensitivity analyses were conducted: (1) aligning disease definitions with the WHO criteria for T2DM and impaired fasting glucose and (2) employing a competing risk model analysis for three possible outcomes

(normoglycaemia, prediabetes, diabetes). Statistical analyses were conducted utilizing R-4.3.0, SAS 9.4, and Empower®2.0. $P < 0.05$ was considered to indicate statistical significance.

RESULTS

Participant Characteristics

Table 1 outlines the characteristics of the study participants, divided according to TyG index quartiles, while **Table 2** shows the same for the TG/HDL-C ratio quartiles. Among the 15,012 prediabetic subjects, the median values for the TyG index and TG/HDL-C ratio were 8.81 (IQR 8.41-9.21) and 2.51 (IQR 1.58-3.93), respectively. An increase in the two indices corresponded with older age, male sex, higher blood pressure, greater BMI, lower levels of TC, TG, HDL-C, and LDL-C, and diminished ALT, AST, and Cr levels. Current alcohol consumption and smoking status were also linked to the two highest baseline indices. The distributions of the two indices are depicted in **Supplementary File 2: Fig. S1**, which shows a normal distribution for the TyG index and a right-skewed distribution for the TG/HDL-C ratio.

Association of Baseline TyG or TG/HDL-C Ratio with Diabetes

Schoenfeld residual plots for the two indices over time(**Supplementary File 2: Fig. S2**) confirmed the validity of the proportional hazards assumption for the Cox proportional hazards model. After testing for collinearity, LDL-C, TG, and TC were omitted from further multivariable models due to a variance inflation factor above the threshold of 5, suggesting multicollinearity (**Supplementary File 1: Table S1**).

Over a median follow-up duration of 2.87 years (IQR 2.08 – 3.57; totaling 34,268 person-years), 1,730 (11.5%) prediabetes patients developed type 2 diabetes. The risk of diabetes increased with increasing TyG index values, even after adjusting for sociodemographic variables. The adjusted risk ratios for diabetes in relation to the lowest quartile for both indices showed a progressive increase from quartile Q2 to Q4 for TyG (1.52, 95% CI: 1.28 – 1.81 to 2.59, 95% CI: 2.20 – 3.05) and for the TG/HDL-C ratio (1.31, 95% CI: 1.11 – 1.55 to 2.03, 95% CI: 1.71 – 2.40). This indicates a significant association between higher quartiles of these indices and the risk of diabetes. Significant trends were observed across quartiles for both indices (P <0.001). Notably, for every one SD increase in the logarithmically transformed TyG index, the likelihood of developing diabetes increased by 23%. Similarly, the risk increased by 43% for each increase in the TG/HDL-C ratio. (**Table 3**). Multivariate adjusted analysis using limited cubic spline revealed a linear correlation between the progression to diabetes and the two indices in question (TyG index: p = 0.031; TG/HDL-C index: P <0.001) (**Fig. 2**).

Ability of Both indices for Assessing Diabetes Risk

In the diabetes risk prediction model, the AUCs for the TyG index and the TG/HDL-C ratio were 0.726 (95% CI: 0.717-0.735) and 0.710 (95% CI: 0.698-0.719), respectively, with a notable difference between them (P = 0.03) (Fig. 3). The positive and negative likelihood ratios for the TyG index were 1.756 and 0.323, respectively, and for the TG/HDL-C ratio, they were 1.824 and 0.442, respectively (Supplementary File 1: Table S2).

In the diabetes risk prediction model, the AUCs for the TyG index and the TG/HDL-C ratio were 0.726 (95% CI: 0.717-0.735) and 0.710 (95% CI: 0.698-0.719), respectively, with significant differences between them (*P* = 0.03) (**Fig. 3**). The positive and negative likelihood ratios for the TyG index were 1.756 and 0.323, respectively. Similarly, the TG/HDL-C ratios were 1.824 and 0.442, respectively (**Supplementary File 1: Table S2**).

Subgroup Analysis and Sensitivity Analysis

Stratification variables included age, sex, BMI, and family history of diabetes. The relationship between glycemic deterioration and the TyG index was found to be influenced by age and sex (P for interaction < 0.05). Subgroup analyses by family history and BMI, revealed no significant interaction between the TyG index and the risk of diabetes ($P > 0.05$). Notably, the TyG index was not associated with an increased risk of diabetes among participants with a family history of the disease (HR:1.33, 95% CI: 0.84 – 2.12). The TG/HDL-C ratio exhibited analogous results. (**Table 4**). Competing risk model analysis and sensitivity analyses using the 1999 WHO criteria confirmed the stability of the relationship between the two indices and glycemic deterioration (**Supplementary File 1: Table S3**), demonstrating a significant correlation ($P < 0.001$).

DISCUSSION

In this longitudinal, multicenter health examination cohort of adult prediabetes patients, high initial TyG index and TG/HDL-C ratio were identified as significant predictors of diabetes development. The correlation remained robust after accounting for variables that could confound the results. When comparing the highest quartile to the lowest quartile, there was a significant increase in the risk of diabetes, with a 2.03-fold increase in the TyG index and a 2.59-fold increase in the TG/HDL-C ratio greater risk. Moreover, by employing smooth curve fitting techniques, a linear correlation was observed between both indices and diabetes risk, with the TyG index demonstrating superior predictive capabilities. Sensitivity analysis revealed consistent results, and subgroup analyses indicated that age and sex were influential modifiers of the disease.

Previous studies have demonstrated that individuals with prediabetes have an

increased likelihood of developing diabetes [22, 23], findings consistent with our findings. Over a median follow-up period of 2.87 years, we observed that 11.5% of the prediabetic patients developed diabetes. Diabetes primarily develops as a result of IR [24]. Research has shown a correlation between the risk of diabetes and both indices, which are frequently used as proxy indicators of IR [25-28]. Furthermore, elevated levels of the two indices have also been correlated with increased prediabetes risk [13, 29]. However, there is a dearth of research exploring the relationship between these indices and the risk of diabetes progression, especially among prediabetic individuals [16,30,31]. After adjusting for potential confounding factors, previous works revealed a positive correlation between the two parameters and diabetes risk. The current findings agree with those of earlier investigations; the analysis based on smooth curve fitting revealed an approximately linear correlation between the two parameters and progression to diabetes. Additionally, the present work demonstrated a positive correlation between the TyG index and progression to diabetes. Moreover, sensitivity analysis demonstrated the reliability of the two indices in predicting the risk of diabetes progression. These findings strengthen the body of research on the two markers of prediabetic glucose state transition risk and highlight their value as practical early indicators of subclinical disease progression.

The current analysis revealed that the TyG index exhibits a superior ability to predict diabetes risk compared to TG/HDL-C, indicating that the TyG index may be a more useful predictor of T2DM, but the underlying mechanism remains unresolved. Higher TyG index values are linked to a greater likelihood of diabetes development in prediabetic patients, which may be attributed to IR [11, 32]. In accordance with Romero et al.'s study [11], we hypothesized that the connection between the two parameters and diabetes risk is mediated by IR. Since IR can lead to aberrant blood glucose levels, this may explain why the two indices have a robust predictive capacity for prediabetes. Additionally, pancreatic beta-cell dysfunction could account for the association between these two indices and disease progression [33, 34]. Beta cells are susceptible to glucotoxicity and lipotoxicity, and it is well established that elevated glucose levels increase reactive oxygen species that inflict cellular damage [35]. Furthermore, increased triglyceride levels raise ceramide and nitric oxide levels, which in turn cause beta-cell death and IR in response to glucose. Moreover, low HDL-C levels inhibit cholesterol efflux, leading to its accumulation in beta cells, causing beta-cell malfunction, elevated blood sugar, reduced insulin production, and eventual beta-cell loss [36]. Collectively, these mechanisms may underlie the correlation between the two parameters and the risk of diabetes [37].

Strengths and limitations

The strengths of this study lie in its detailed examination of the nonlinear relationship between the two indices and diabetes risk among the prediabetic population, its use of ROC analysis to evaluate the predictive ability, and its sensitivity analyses that consider various methodological approaches. Additionally, it includes a subgroup analysis to account for potential confounding factors and employs established quality control methods for measuring study variables. However,

this work has several limitations. First, the findings are based on FPG due to database limitations; the absence of information on oral glucose tolerance tests and glycated haemoglobin may lead to an underestimation of the incidence of diabetes. However, since nondiabetic individuals seldom undergo oral glucose tolerance testing, a diagnosis based on FPG may be sufficiently accurate for representing affected individuals. Second, the relatively short median follow-up period that may not adequately reflect the risk relationship. Third, the study population was limited, and further research is required to enhance the identification and prognosis of diabetes development in other countries and ethnic groups. Fourth, residual confounding due to unavailable data on diet, physical activity, certain medical conditions, or metabolic parameters may introduce unobserved variables even after comprehensive adjustment. Fifth, the present study did not include nonalcoholic fatty liver disease (NAFLD), which is prevalent among prediabetic patients and could influence the findings. Finally, the study evaluated only the baseline indices, and tracking long-term changes could provide additional insights.

CONCLUSION

This study underscores the association between higher initial TyG or TG/HDL-C levels and heightened T2DM risk in prediabetic individuals. The TyG index, in particular, shows superior predictive power for diabetes risk, suggesting that efforts to prevent the onset of diabetes may benefit from targeting the reduction in TyG levels in patients with prediabetes.

oSR83p7TSG

ORIGINALITY REPORT

18%

SIMILARITY INDEX

PRIMARY SOURCES

1	www.ncbi.nlm.nih.gov<br>Internet	115 words — 4%
2	www.science.gov<br>Internet	70 words — 2%
3	Yanfei Sun, Zhibin Wang, Zhiqiang Huang, Haofei Hu, Yong Han. "The Association Between the Triglyceride-to-High-Density Lipoprotein Cholesterol Ratio and the Risk of Progression to Diabetes From Prediabetes: A 5-year Cohort Study in Chinese Adults", Frontiers in Endocrinology, 2022<br>Crossref	43 words — 1%
4	Linhao Zhang, Ling Zeng. "Non-linear association of triglyceride-glucose index with prevalence of prediabetes and diabetes: a cross-sectional study", Frontiers in Endocrinology, 2023<br>Crossref	33 words — 1%
5	Haipeng Yao, Zhen Sun, Wei Yuan, Chen Shao, Honghua Cai, Lihua Li, Yongjiang Qian, Zhongqun Wang. "Relationship Between the Triglyceride-Glucose Index and Type 2 Diabetic Macroangiopathy: A Single-Center Retrospective Analysis", Diabetes, Metabolic Syndrome and Obesity: Targets and Therapy, 2022<br>Crossref	25 words — 1%

6	www.researchgate.net<br>Internet	25 words — 1%
7	Kang Wang, Zhangling Chen, Maoqi Shen, Pengfei Chen et al. "Dietary fruits and vegetables and risk of cardiovascular diseases in elderly Chinese", European Journal of Public Health, 2023<br>Crossref	18 words — 1%
8	cardiab.biomedcentral.com<br>Internet	18 words — 1%
9	Zhang, Liying, Shanying Chen, Aiwen Deng, Xinyu Liu, Yan Liang, Xiaofei Shao, Mingxia Sun, and Hequn Zou. "Association between Lipid Ratios and Insulin Resistance in a Chinese Population", PLoS ONE, 2015.<br>Crossref	17 words — 1%
10	worldwidescience.org<br>Internet	17 words — 1%
11	Jialei Li, Kexin Wang, Song Li, Pengsheng Wu, Xiangpeng Wang, Yi He, Wenrui Tang. "Clinical study of multifactorial diagnosis in prostate biopsy", The Prostate, 2023<br>Crossref	16 words — 1%
12	www.aginganddisease.org<br>Internet	15 words — 1%
13	Dimitrios Kazantzis, Christopher Holmes, Naomi Wijesingha, Sobha Sivaprasad. "Changes in foveal avascular zone parameters in individuals with prediabetes compared to normoglycemic controls: a systematic review and meta-analysis", Eye, 2024<br>Crossref	12 words — < 1%

14 Hailun Qin, Zekai Chen, Yunzhang Zhang, Lingyu Wang, Piao Ouyang, Lan Cheng, Yonggang Zhang. "Triglyceride to high-density lipoprotein cholesterol ratio is associated with incident diabetes in men: A retrospective study of Chinese individuals", Journal of Diabetes Investigation, 2019 — 12 words — < 1%
Crossref

---

15 lipidworld.biomedcentral.com — 12 words — < 1%
Internet

---

16 Panya Chamroonkiadtikun, Thareerat Ananchaisarp, Worawit Wanichanon. "The triglyceride-glucose index, a predictor of type 2 diabetes development: A retrospective cohort study", Primary Care Diabetes, 2020 — 11 words — < 1%
Crossref

---

17 www.jstage.jst.go.jp — 11 words — < 1%
Internet

---

18 Tianyu Li, Deshan Yuan, Peizhi Wang, Guyu Zeng et al. "Association of prognostic nutritional index level and diabetes status with the prognosis of coronary artery disease: a cohort study", Diabetology & Metabolic Syndrome, 2023 — 9 words — < 1%
Crossref

---

19 pubmed.ncbi.nlm.nih.gov — 9 words — < 1%
Internet

---

20 Kirsti Vik Hjerkind, Jo S Stenehjem, Tom I L Nilsen. "Adiposity, physical activity and risk of diabetes mellitus: prospective data from the population-based HUNT study, Norway", BMJ Open, 2017 — 8 words — < 1%
Crossref

21	journals.sagepub.com<br>Internet	8 words — < 1%
22	www.dovepress.com<br>Internet	8 words — < 1%
23	www.frontiersin.org<br>Internet	8 words — < 1%
24	www.mdpi.com<br>Internet	8 words — < 1%

EXCLUDE QUOTES	OFF	EXCLUDE SOURCES	OFF
EXCLUDE BIBLIOGRAPHY	OFF	EXCLUDE MATCHES	OFF
