# Supplementary material for: A longitudinal study on the impact of the TyG Index and TG/HDL-C ratio on the risk of type 2 diabetes in Chinese patients with prediabetes
Source: Lipids Health Dis. 2024 Aug 22;23:262. doi: 10.1186/s12944-024-02239-1 (PMC11340070; doi:10.1186/s12944-024-02239-1)
Supplement: Supplementary file 7 — Supplementary Material 7 [file 12944_2024_2239_MOESM7_ESM.docx]

**A Longitudinal Study on the Impact of the TyG Index and TG/HDL-C Ratio on the Risk of Type 2 diabetes in Chinese Patients with Prediabetes**

Bo Chen^1,2^, Jingjing Zeng^1,2^, Menglin Fan^1,2^, Qiqi You^1,2^, Chenyue Wang^1,2^, Ke Wang^3,2^, Minghui Qin^4,2*^, Shaoyong Xu^1,2*^

^1^Department of Endocrinology, Xiangyang Central Hospital, Affiliated Hospital of Hubei University of Arts and Science, Xiangyang, Hubei, China.

^2^Center for Clinical Evidence-Based and Translational Medicine, Xiangyang Central Hospital, Affiliated Hospital of Hubei University of Arts and Science, Xiangyang, Hubei, China.

^3^Department of Preventive Medicine, Medical College, Hubei University of Arts and Science, Xiangyang 441053, China.

^4^Department of Traditional Chinese Medicine, Xiangyang Central Hospital, Affiliated Hospital of Hubei University of Art and Science, Xiangyang, Hubei, China.

^*^Correspondence: Shaoyong Xu, E-mail: yoji_xu@hotmail.com; Minghui Qin, E-mail: 1317146615@qq.com

**Abstract**

**Objective:** To elucidate the impact and predictive value of the Triglyceride Glucose Index (TyG) and the ratio of Triglycerides to High-Density Lipoprotein Cholesterol (TG/HDL-C) in identifying the risk of diabetes progression in Chinese individuals with prediabetes.

**Methods:** This longitudinal study enrolled 15,012 prediabetic adults from the Rich Healthcare Group between 2010 and 2016. Diabetes was defined as self-reported diabetes or a fasting glucose level ≥ 7.0 mmol/L. The Cox proportional hazards models was utilized to assess the relationship between the two indices and the risk of developing diabetes. The predictive efficacy of the two markers was gauged by the area under the curve (AUC).

**Results:** Over a median follow-up period of 2.87 years, 1,730 (11.5%) prediabetic participants developed diabetes. The adjusted hazard ratios for the top quartile of the TyG index and the TG/HDL-C ratio were 2.03 (95% confidence interval [CI]: 1.71-2.40) and 2.59 (95% CI: 2.20-3.05), respectively, compared to the lowest quartile. A significant trend of increasing diabetes risk with higher quartiles of both indices was observed. The AUC for the adjusted prediction model for prediabetes-to-diabetes transition was 0.726 for the TyG index and 0.710 for the TG/HDL-C ratio. The difference in AUCs was statistically significant (*P* = 0.03).

**Conclusions:** The baseline TyG index or TG/HDL-C ratio was significantly associated with an increased risk of diabetes in prediabetic individuals. The TyG index demonstrated superior predictive accuracy, underscoring its importance in preventing diabetes in prediabetic individuals.

**Keywords** TyG, Diabetes, TG/HDL-C ratio, Area under curve

**INTRODUCTION**

Prediabetes is a critical health state in which blood glucose levels are higher than typical but do not reach the thresholds required for type 2 diabetes mellitus (T2DM) [1]. The prevalence of prediabetes is increasing globally, with projections estimating it will affect one billion individuals by 2045 [2, 3]. Each year, 5-10% of those with prediabetes progress to T2DM. Prediabetes is linked to an increased risk of macrovascular and microvascular complications, such as stroke, peripheral arterial disease, myocardial infarction and retinopathy, neuropathy and nephropathy [3-5]. Additionally, prediabetes is linked to an elevated risk of all-cause and cardiovascular mortality [6]. Despite these risks, the majority of individuals with prediabetes remain unaware of their metabolic condition, highlighting the critical need for early detection and intervention of modifiable factors.

Insulin resistance (IR) is characterized by a diminished sensitivity to insulin in peripheral tissues [7] and is a pivotal factor in the progression to diabetes. Enhancing β-cell function and insulin sensitivity can stabilize prediabetes and promote a return to normoglycemia. Although the euglycemic-hyperinsulinemic clamp (HEC) is recognized as the benchmark for assessing IR, its high cost and complexity render it impractical for widespread clinical application [8]. Consequently, there is a drive to identify efficient and cost-effective markers. The triglyceride glucose (TyG) index and the triglyceride to high-density lipoprotein cholesterol (TG/HDL-C) ratio have emerged as reliable surrogates for IR [9, 10], given their strong correlation with HEC and their suitability for large-scale epidemiologic studies and clinical practice [11, 12].

Recent studies have established a link between these two parameters and the incidence of prediabetes or diabetes among individuals in the general population [13-15]. However, the precise relationship between these markers and the risk of diabetes progression in prediabetic individuals is not fully understood. To date, only limited research has explored the relationship between the TG/HDL-C ratio and the incidence of diabetes in prediabetic individuals [16], Furthermore, whether the TyG index can accurately predict the advancement to diabetes remains to be determined.

Given the ease and affordability of measuring the TyG and TG/HDL-C ratios, understanding their relationship with disease progression, especially considering the widespread occurrence of prediabetes and its associated complications, could significantly aid in prevention and treatment strategies. This study used multicenter physical examination data from the China Fukang Medical Group to explore the predictive value of these indices for diabetes risk in the prediabetic population.

**METHODS**

**Study Design and Participants**

This retrospective cohort analysis leveraged longitudinal data from the China Rich Medical Group’s multicenter health screening cohort spanning from 2010 to 2016. Eligible participants underwent at least two health screenings within this timeframe. The original cohort, comprising 685,277 Chinese adults over 20 years of age, was established to explore the role of obesity in diabetes development [17]. After applying exclusion criteria consistent with previous study aims, 211,833 participants with documented type 2 diabetes outcomes were selected for analysis. Anonymized data were made available on the Dryad digital repository for secondary analysis. No additional ethical approval was required for subsequent analyses, as the initial result was granted by the Rich Healthcare Subcommittee Review Board. This research adhered to the STROBE guidelines and the Declaration of Helsinki principles.

The study population comprised 26,018 baseline prediabetic individuals, defined by measurements ranging from 5.6 to 6.9 mmol/L, following the American Diabetes Association criteria. The transition from prediabetes to diabetes was the primary dependent variable, while the TyG index and TG/HDL-C ratio served as independent variables. Participants with abnormal (n=412) or missing (n=10,594) data for these indices were excluded, yielding a final sample size of 15,012 participants. The process of subject inclusion and exclusion is illustrated in **Figure 1**.

**Baseline Indicator Measurement Assessment**

In the initial phase of the study, trained investigators systematically collected demographic data, including age and sex, as well as lifestyle factors such as alcohol consumption and smoking, and family history of diabetes using a self-administered questionnaire. Smoking status was ascertained by asking participants, "Do you smoke?" with options. Those who reported never smoking were classified as “never smokers”. Former smokers were identified as those who had smoked but since quit smoking, while current smokers were those who continued smoking. Alcohol consumption was determined by inquiring, "Have you consumed any alcoholic beverages in the past year?" Nondrinkers were defined as individuals who had not consumed alcohol, while current drinkers were those who continued to consume alcohol daily. Former drinkers were those who had ceased alcohol consumption. Precision was applied in recording anthropometric data including body weight and height, which allowed for the subsequent computation of the body mass index (BMI). Concurrently, blood pressure was measured utilizing a mercury sphygmomanometer.

Fasting blood samples were obtained after at least 10 hours of fasting and processed within 2 hours of collection. A Beckman 5800 automatic biochemical analyser was used to assess common biochemical markers, including TG, total cholesterol (TC), fasting plasma glucose (FPG), HDL-C, low-density lipoprotein cholesterol (LDL-C), blood urea nitrogen (BUN), creatinine (Cr), alanine aminotransferase (ALT), and aspartate aminotransferase (AST).

**Variables**

The calculation of the TyG index was performed utilizing the specific formula. TyG Index = Ln ((fasting plasma glucose (mmol/L)×18) × (triglyceride (mmol/L)×88.5)/2) [11], and the TG/HDL-C ratio was determined by another formula: TG/HDL-c ratio = (triglyceride (mmol/L) ×88.5)/(high-density lipoprotein cholesterol (mmol/L) ×38.67) [18].

**Outcome Measures**

The primary outcome was the progression from prediabetes to diabetes, with prediabetes defined by baseline FPG levels between 5.6 and 6.9 mmol/L. Diabetes was identified through self-reports or an FPG level of concentration of 7.0 mmol/L or higher at the final visit [19].

**Statistical analysis**

To mitigate bias from missing data, we utilized multiple imputations based on chained equations to estimate missing values. Continuous variables are presented as median values alongside interquartile ranges (IQRs) or means accompanied by their standard deviations (SDs). In contrast, categorical variables are expressed in terms of frequency, marked as a percentage. To evaluate the significant differences among the quartiles of the TyG index or the TG/HDL-C ratio, methods such as chi-square tests, Kruskal‒Wallis tests, and variance analysis were utilized.

Collinearity between the two indices and other covariates was diagnosed using tolerance and variance inflation factors. The associations between either the TyG index or the TG/HDL-C ratio and the risk of diabetes were gauged using the Cox proportional hazards model, with adjustments for various covariates. Prior to modelling, the assumption of proportional hazards was verified through Schoenfeld residuals. Model 1 was unadjusted, while Model 2 included adjustments for sex, age, HDL-C, LDL-C, AST, ALT, blood pressure, BMI, Cr, BUN, alcohol consumption status, smoking status,, and familial diabetes history. Linear trends were discerned through the median score for each quartile, and the impact of a standard deviation increase in the index on the outcome risk was evaluated as a continuous variable. Nonlinear correlation were explored using a fully adjusted Cox model with smooth curve fitting functions. The predictive efficacy of the two indices was assessed via the area under the curve (AUC) from receiver operator characteristic (ROC) curve analysis.

Potential heterogeneity in the relationship between the two markers and the risk of diabetes was investigated across prevalent phenotypes, including sex, diabetes family history, BMI categories [20], and age groups based on WHO criteria [21]. Interactions among groups were compared using likelihood ratio tests.

To ensure robustness, four sensitivity analyses were conducted: (1) aligning disease definitions with the WHO criteria for T2DM and impaired fasting glucose; (2) employing a competing risk model analysis for three possible outcomes (normoglycaemia, prediabetes, diabetes); (3) Conducting an analysis on the original dataset without multiple imputation; (4) Adjusting for the interaction of sex or age with the TyG index or the TG/HDL-C ratio in the Cox proportional hazards model. Statistical analyses were conducted utilizing R-4.3.0, SAS 9.4, and Empower®2.0. *P* < 0.05 was considered to indicate statistical significance.

**RESULTS**

**Participant Characteristics**

**Table 1** outlines the characteristics of the study participants, divided according to TyG index quartiles, while **Table 2** shows the same for the TG/HDL-C ratio quartiles. Among the 15,012 prediabetic subjects, the median values for the TyG index and TG/HDL-C ratio were 8.81 (IQR 8.41-9.21) and 2.51 (IQR 1.58-3.93), respectively. An increase in the two indices corresponded with older age, male sex, higher blood pressure, greater BMI, lower levels of TC, TG, HDL-C, and LDL-C, and diminished ALT, AST, and Cr levels. Current alcohol consumption and smoking status were also linked to the two highest baseline indices. The distributions of the two indices are depicted in **Supplementary File 2: Fig. S1**, which shows a normal distribution for the TyG index and a right-skewed distribution for the TG/HDL-C ratio.

**Association of Baseline TyG or TG/HDL-C Ratio with Diabetes**

Schoenfeld residual plots for the two indices over time(**Supplementary File 2: Fig. S2**) confirmed the validity of the proportional hazards assumption for the Cox proportional hazards model. After testing for collinearity, LDL-C, TG, and TC were omitted from further multivariable models due to a variance inflation factor above the threshold of 5, suggesting multicollinearity (**Supplementary File 1: Table S1**).

Over a median follow-up duration of 2.87 years (IQR 2.08–3.57; totaling 34,268 person-years), 1,730 (11.5%) prediabetes patients developed type 2 diabetes. The risk of diabetes increased with increasing TyG index values, even after adjusting for sociodemographic variables. The adjusted risk ratios for diabetes in relation to the lowest quartile for both indices showed a progressive increase from quartile Q2 to Q4 for TyG (1.52, 95% CI: 1.28–1.81 to 2.59, 95% CI: 2.20–3.05) and for the TG/HDL-C ratio (1.31, 95% CI: 1.11–1.55 to 2.03, 95% CI: 1.71–2.40). This indicates a significant association between higher quartiles of these indices and the risk of diabetes. Significant trends were observed across quartiles for both indices (*P* <0.001). Notably, for every one SD increase in the logarithmically transformed TyG index, the likelihood of developing diabetes increased by 23%. Similarly, the risk increased by 43% for each increase in the TG/HDL-C ratio. (**Table 3**). Multivariate adjusted analysis using limited cubic spline revealed a linear correlation between the progression to diabetes and the two indices in question (TyG index: p = 0.031; TG/HDL-C index: *P* <0.001) (**Fig. 2**).

**Ability of** **Both indices for** **Assessing** **Diabetes Risk**

In the diabetes risk prediction model, the AUCs for the TyG index and the TG/HDL-C ratio were 0.726 (95% CI: 0.717-0.735) and 0.710 (95% CI: 0.698-0.719), respectively, with a notable difference between them (P = 0.03) (Fig. 3). The positive and negative likelihood ratios for the TyG index were 1.756 and 0.323, respectively, and for the TG/HDL-C ratio, they were 1.824 and 0.442, respectively (**Supplementary File 1: Table S2**).

In the diabetes risk prediction model, the AUCs for the TyG index and the TG/HDL-C ratio were 0.726 (95% CI: 0.717-0.735) and 0.710 (95% CI: 0.698-0.719), respectively, with significant differences between them (*P* = 0.03) (**Fig. 3**). The positive and negative likelihood ratios for the TyG index were 1.756 and 0.323, respectively. Similarly, the TG/HDL-C ratios were 1.824 and 0.442, respectively **(Supplementary File 1: Table S2**).

**Subgroup Analysis and Sensitivity Analysis**

Stratification variables included age, sex, BMI, and family history of diabetes. The relationship between glycemic deterioration and the TyG index was found to be influenced by age and sex (*P* for interaction < 0.05). Subgroup analyses by family history and BMI, revealed no significant interaction between the TyG index and the risk of diabetes (*P* > 0.05). Notably, the TyG index was not associated with an increased risk of diabetes among participants with a family history of the disease (HR:1.33, 95% CI: 0.84–2.12). The TG/HDL-C ratio exhibited analogous results. (**Table 4**). Competing risk model analysis, the original dataset analysis without multiple imputation, adjusting for the interaction of sex or age with the two indices, and sensitivity analyses using the 1999 WHO criteria definition confirmed the stability of the relationship between the two indices and glycemic deterioration (**Supplementary File 1: Table S3**), demonstrating a significant correlation (*P* < 0.001).

**DISCUSSION**

In this longitudinal, multicenter health examination cohort of adult prediabetes patients, high initial TyG index and TG/HDL-C ratio were identified as significant predictors of diabetes development. The correlation remained robust after accounting for variables that could confound the results. When comparing the highest quartile to the lowest quartile, there was a significant increase in the risk of diabetes, with a 2.03-fold increase in the TyG index and a 2.59-fold increase in the TG/HDL-C ratio greater risk. Moreover, by employing smooth curve fitting techniques, a linear correlation was observed between both indices and diabetes risk, with the TyG index demonstrating superior predictive capabilities. Sensitivity analysis revealed consistent results, and subgroup analyses indicated that age and sex were influential modifiers of the disease.

Previous studies have demonstrated that individuals with prediabetes have an increased likelihood of developing diabetes [22, 23], findings consistent with our findings. Over a median follow-up period of 2.87 years, we observed that 11.5% of the prediabetic patients developed diabetes. Diabetes primarily develops as a result of IR [24]. Research has shown a correlation between the risk of diabetes and both indices, which are frequently used as proxy indicators of IR [25-28]. Furthermore, elevated levels of the two indices have also been correlated with increased prediabetes risk [13, 29]. However, there is a dearth of research exploring the relationship between these indices and the risk of diabetes progression, especially among prediabetic individuals [16,30,31]. After adjusting for potential confounding factors, previous works revealed a positive correlation between the two parameters and diabetes risk. The current findings agree with those of earlier investigations; the analysis based on smooth curve fitting revealed an approximately linear correlation between the two parameters and progression to diabetes. Additionally, the present work demonstrated a positive correlation between the TyG index and progression to diabetes. Moreover, sensitivity analysis demonstrated the reliability of the two indices in predicting the risk of diabetes progression. These findings strengthen the body of research on the two markers of prediabetic glucose state transition risk and highlight their value as practical early indicators of subclinical disease progression.

The current analysis revealed that the TyG index exhibits a superior ability to predict diabetes risk compared to TG/HDL-C, indicating that the TyG index may be a more useful predictor of T2DM, but the underlying mechanism remains unresolved. Higher TyG index values are linked to a greater likelihood of diabetes development in prediabetic patients, which may be attributed to IR [11, 32]. In accordance with Romero et al.'s study [11], we hypothesized that the connection between the two parameters and diabetes risk is mediated by IR. Since IR can lead to aberrant blood glucose levels, this may explain why the two indices have a robust predictive capacity for prediabetes. Additionally, pancreatic beta-cell dysfunction could account for the association between these two indices and disease progression [33, 34]. Beta cells are susceptible to glucotoxicity and lipotoxicity, and it is well established that elevated glucose levels increase reactive oxygen species that inflict cellular damage [35]. Furthermore, increased triglyceride levels raise ceramide and nitric oxide levels, which in turn cause beta-cell death and IR in response to glucose. Moreover, low HDL-C levels inhibit cholesterol efflux, leading to its accumulation in beta cells, causing beta-cell malfunction, elevated blood sugar, reduced insulin production, and eventual beta-cell loss [36]. Collectively, these mechanisms may underlie the correlation between the two parameters and the risk of diabetes [37].

**Strengths and limitations**

The strengths of this study lie in its detailed examination of the nonlinear relationship between the two indices and diabetes risk among the prediabetic population, its use of ROC analysis to evaluate the predictive ability, and its sensitivity analyses that consider various methodological approaches. Additionally, it includes a subgroup analysis to account for potential confounding factors and employs established quality control methods for measuring study variables. However, this work has several limitations. First, the findings are based on FPG due to database limitations; the absence of information on oral glucose tolerance tests and glycated haemoglobin may lead to an underestimation of the incidence of diabetes. However, since nondiabetic individuals seldom undergo oral glucose tolerance testing, a diagnosis based on FPG may be sufficiently accurate for representing affected individuals. Second, the relatively short median follow-up period that may not adequately reflect the risk relationship. Third, the study population was limited, and further research is required to enhance the identification and prognosis of diabetes development in other countries and ethnic groups. Fourth, residual confounding due to unavailable data on diet, physical activity, certain medical conditions, or metabolic parameters may introduce unobserved variables even after comprehensive adjustment. Fifth, the present study did not include nonalcoholic fatty liver disease (NAFLD), which is prevalent among prediabetic patients and could influence the findings. Finally, the study evaluated only the baseline indices, and tracking long-term changes could provide additional insights.

**CONCLUSION**

This study underscores the association between higher initial TyG or TG/HDL-C levels and heightened T2DM risk in prediabetic individuals. The TyG index, in particular, shows superior predictive power for diabetes risk, suggesting that efforts to prevent the onset of diabetes may benefit from targeting the reduction in TyG levels in patients with prediabetes.

**Acknowledgements**

Not applicable.

**Authors contributions**

Bo Chen, Shaoyong Xu, and Minghui Qin contributed to the study concept and design, researched and interpreted the data, and drafted the manuscript. Bo Chen, Jingjing Zeng, Menglin Fan, Qiqi You, Chenyue Wang, and Ke Wang analyzed the data and reviewed the manuscript. All authors read and approved the final manuscript.

**Funding**

The study was partly supported by the Young Talents Project of Hubei Provincial Health Commission, China (Grand number WJ2021Q012); Science and Technology Research Key Project of Education Department of Hubei Province, China (Grand number D20212602); Xiangyang Science and Technology Plan Project, China (No. 2022YL08B); and The Hubei Provincial Natural Science Foundation of China (No. 2022CFC032).

**Data Availability**

The raw data can be downloaded from the ‘DATADRYAD’ database (www. Datadryad.org). Dryad Digital Rep. <https://datadryad.org/stash/dataset/doi:10.5061/dryad.ft8750v>.

**Declarations**

**Competing interests**

The authors declare that they have no competing interests.

**Ethics approval and consent to participate**

The original study followed guidelines outlined by the Helsinki Declaration and was approved by the Rich Healthcare Group Review Board. In addition, the Rich Healthcare Group Review Board has waived informed consent for the current retrospective study. All methods were performed in accordance with the relevant the Declaration of Helsinki.

**Consent for publication**

Not applicable.

**Reference**

1. Edwards CM, Cusi K: **Prediabetes: A Worldwide Epidemic**. *Endocrinology and metabolism clinics of North America* 2016, **45**(4):751-764.

2. Sun H, Saeedi P, Karuranga S, Pinkepank M, Ogurtsova K, Duncan BB, Stein C, Basit A, Chan JCN, Mbanya JC *et al*: **IDF Diabetes Atlas: Global, regional and country-level diabetes prevalence estimates for 2021 and projections for 2045**. *Diabetes research and clinical practice* 2022, **183**:109119.

3. Rooney MR, Fang M, Ogurtsova K, Ozkan B, Echouffo-Tcheugui JB, Boyko EJ, Magliano DJ, Selvin E: **Global Prevalence of Prediabetes**. *Diabetes care* 2023, **46**(7):1388-1394.

4. Qiu S, Cai X, Yuan Y, Xie B, Sun Z, Wang D, Wu T: **Muscle strength and prediabetes glycemic worsening and regression in middle-aged and older adults: a prospective cohort study**. *Journal of cachexia, sarcopenia and muscle* 2022, **13**(2):909-918.

5. Tabák AG, Herder C, Rathmann W, Brunner EJ, Kivimäki M. Prediabetes: a high-risk state for diabetes development. Lancet. 2012,379(9833):2279-90.

6. Huang Y, Cai X, Mai W, Li M, Hu Y. Association between prediabetes and risk of cardiovascular disease and all cause mortality: systematic review and meta-analysis. BMJ. 2016 Nov 23;355:i5953. doi: 10.1136/bmj.i5953. PMID: 27881363; PMCID: PMC5121106.

7. Færch K, Vistisen D, Pacini G, Torekov SS, Johansen NB, Witte DR, Jonsson A, Pedersen O, Hansen T, Lauritzen T *et al*: **Insulin Resistance Is Accompanied by Increased Fasting Glucagon and Delayed Glucagon Suppression in Individuals With Normal and Impaired Glucose Regulation**. *Diabetes* 2016, **65**(11):3473-3481.

8. Bonora E, Targher G, Alberiche M, Bonadonna RC, Saggiani F, Zenere MB, Monauni T, Muggeo M: **Homeostasis model assessment closely mirrors the glucose clamp technique in the assessment of insulin sensitivity: studies in subjects with various degrees of glucose tolerance and insulin sensitivity**. *Diabetes care* 2000, **23**(1):57-63.

9. Darshan An V, Rajput R, Meena, Mohini, Garg R, Saini S: **Comparison of triglyceride glucose index and HbA1C as a marker of prediabetes - A preliminary study**. *Diabetes & metabolic syndrome* 2022, **16**(9):102605.

10. Er LK, Wu S, Chou HH, Hsu LA, Teng MS, Sun YC, Ko YL: **Triglyceride Glucose-Body Mass Index Is a Simple and Clinically Useful Surrogate Marker for Insulin Resistance in Nondiabetic Individuals**. *PloS one* 2016, **11**(3):e0149731.

11. Guerrero-Romero F, Simental-Mendía LE, González-Ortiz M, Martínez-Abundis E, Ramos-Zavala MG, Hernández-González SO, Jacques-Camarena O, Rodríguez-Morán M: **The product of triglycerides and glucose, a simple measure of insulin sensitivity. Comparison with the euglycemic-hyperinsulinemic clamp**. *The Journal of clinical endocrinology and metabolism* 2010, **95**(7):3347-3351.

12. Giannini C, Santoro N, Caprio S, Kim G, Lartaud D, Shaw M, Pierpont B, Weiss R: **The triglyceride-to-HDL cholesterol ratio: association with insulin resistance in obese youths of different ethnic backgrounds**. *Diabetes care* 2011, **34**(8):1869-1874.

13. Wen J, Wang A, Liu G, Wang M, Zuo Y, Li W, Zhai Q, Mu Y, Gaisano HY, He Y *et al*: **Elevated triglyceride-glucose (TyG) index predicts incidence of Prediabetes: a prospective cohort study in China**. *Lipids in health and disease* 2020, **19**(1):226.

14. Low S, Khoo KCJ, Irwan B, Sum CF, Subramaniam T, Lim SC, Wong TKM: **The role of triglyceride glucose index in development of Type 2 diabetes mellitus**. *Diabetes research and clinical practice* 2018, **143**:43-49.

15. Park HM, Lee HS, Lee YJ, Lee JH: **The triglyceride-glucose index is a more powerful surrogate marker for predicting the prevalence and incidence of type 2 diabetes mellitus than the homeostatic model assessment of insulin resistance**. *Diabetes research and clinical practice* 2021, **180**:109042.

16. Sun Y, Wang Z, Huang Z, Hu H, Han Y: **The Association Between the Triglyceride-to-High-Density Lipoprotein Cholesterol Ratio and the Risk of Glycemic worsening to Diabetes From Prediabetes: A 5-year Cohort Study in Chinese Adults**. *Frontiers in endocrinology* 2022, **13**:947157.

17. Chen Y, Zhang XP, Yuan J, Cai B, Wang XL, Wu XL, Zhang YH, Zhang XY, Yin T, Zhu XH *et al*: **Association of body mass index and age with incident diabetes in Chinese adults: a population-based cohort study**. *BMJ open* 2018, **8**(9):e021768.

18. Che B, Zhong C, Zhang R, Pu L, Zhao T, Zhang Y, Han L. **Triglyceride-glucose index and triglyceride to high-density lipoprotein cholesterol ratio as potential cardiovascular disease risk factors: an analysis of UK biobank data**. Cardiovasc Diabetol. 2023, 22(1):34.

19. Yang H, Kuang M, Yang R, Xie G, Sheng G, Zou Y. **Evaluation of the role of atherogenic index of plasma in the reversion from Prediabetes to normoglycemia or progression to Diabetes: a multi-center retrospective cohort study**. Cardiovasc Diabetol. 2024, 6;23(1):17.

20. Cen J, Han Y, Liu Y, Hu H: **Evaluated Glomerular Filtration Rate Is Associated With Non-alcoholic Fatty Liver Disease: A 5-Year Longitudinal Cohort Study in Chinese Non-obese People**. *Frontiers in nutrition* 2022, **9**:916704.

21. **World Health Day 2012 focuses on ageing and health**. *Eastern Mediterranean health journal = La revue de sante de la Mediterranee orientale = al-Majallah al-sihhiyah li-sharq al-mutawassit* 2012, **18**(4):303.

22. Heianza Y, Hara S, Arase Y, Saito K, Fujiwara K, Tsuji H, Kodama S, Hsieh SD, Mori Y, Shimano H *et al*: **HbA1c 5·7-6·4% and impaired fasting plasma glucose for diagnosis of prediabetes and risk of glycemic worsening to diabetes in Japan (TOPICS 3): a longitudinal cohort study**. *Lancet (London, England)* 2011, **378**(9786):147-155.

23. Lipska KJ, Inzucchi SE, Van Ness PH, Gill TM, Kanaya A, Strotmeyer ES, Koster A, Johnson KC, Goodpaster BH, Harris T *et al*: **Elevated HbA1c and fasting plasma glucose in predicting diabetes incidence among older adults: are two better than one?** *Diabetes care* 2013, **36**(12):3923-3929.

24. Hulman A, Simmons RK, Brunner EJ, Witte DR, Færch K, Vistisen D, Ikehara S, Kivimaki M, Tabák AG: **Trajectories of glycaemia, insulin sensitivity and insulin secretion in South Asian and white individuals before diagnosis of type 2 diabetes: a longitudinal analysis from the Whitehall II cohort study**. *Diabetologia* 2017, **60**(7):1252-1260.

25. Rong L, Hou N, Hu J, Gong Y, Yan S, Li C, Yang Z, Sun B: **The role of TyG index in predicting the incidence of diabetes in Chinese elderly men: a 20-year retrospective study**. *Frontiers in endocrinology* 2023, **14**:1191090.

26. Park B, Lee HS, Lee YJ: **Triglyceride glucose (TyG) index as a predictor of incident type 2 diabetes among nonobese adults: a 12-year longitudinal study of the Korean Genome and Epidemiology Study cohort**. *Translational research : the journal of laboratory and clinical medicine* 2021, **228**:42-51.

27. Liu H, Liu J, Liu J, Xin S, Lyu Z, Fu X: **Triglyceride to High-Density Lipoprotein Cholesterol (TG/HDL-C) Ratio, a Simple but Effective Indicator in Predicting Type 2 Diabetes Mellitus in Older Adults**. *Frontiers in endocrinology* 2022, **13**:828581.

28. Tohidi M, Asgari S, Chary A, Safiee S, Azizi F, Hadaegh F: **Association of triglycerides to high-density lipoprotein cholesterol ratio to identify future prediabetes and type 2 diabetes mellitus: over one-decade follow-up in the Iranian population**. *Diabetology & metabolic syndrome* 2023, **15**(1):13.

29. Wu L, Wu X, Hu H, Wan Q: **Association between triglyceride-to-high-density lipoprotein cholesterol ratio and prediabetes: a cross-sectional study in Chinese non-obese people with a normal range of low-density lipoprotein cholesterol**. *Journal of translational medicine* 2022, **20**(1):484.

30. Han Y, Hu H, Li Q, Deng Z, Liu D. **Triglyceride glucose-body mass index and the risk of progression to diabetes from prediabetes: A 5-year cohort study in Chinese adults**. Front Public Health. 2023,11:1028461.

31. Sharafi M, Amiri Z, Pezeshki B, Mohsenpour MA, Eftekhari MH, Afrashteh S, Haghjoo E, Farhadi A, Khaleghi M, Mastaneh Z. **Predictive value of triglycerides to high-density lipoprotein cholesterol and triglyceride glycemic index for diabetes incidence in pre-diabetes patients: a prospective cohort study**. J Health Popul Nutr. 2023,42(1):67.

32. Uruska A, Zozulinska-Ziolkiewicz D, Niedzwiecki P, Pietrzak M, Wierusz-Wysocka B: **TG/HDL-C ratio and visceral adiposity index may be useful in assessment of insulin resistance in adults with type 1 diabetes in clinical practice**. *Journal of clinical lipidology* 2018, **12**(3):734-740.

33. Zhou M, Li Z, Min R, Dong Y, Sun Q, Li Y: **Log (TG)/HDL-C ratio as a predictor of decreased islet beta cell function in patients with type 2 diabetes: 6-year cohort study**. *Journal of diabetes* 2015, **7**(5):689-698.

34. Chen Z, Wen J: **Elevated triglyceride-glucose (TyG) index predicts impaired islet β-cell function: A hospital-based cross-sectional study**. *Frontiers in endocrinology* 2022, **13**:973655.

35. Robertson RP, Harmon J, Tran PO, Poitout V. **Beta-cell glucose toxicity, lipotoxicity, and chronic oxidative stress in type 2 diabetes**. Diabetes. 2004 Feb;53 Suppl 1:S119-24.

36. Sorci-Thomas MG, Thomas MJ: **High density lipoprotein biogenesis, cholesterol efflux, and immune cell function**. *Arteriosclerosis, thrombosis, and vascular biology* 2012, **32**(11):2561-2565.

37. Shimodaira M, Niwa T, Nakajima K, Kobayashi M, Hanyu N, Nakayama T: **Serum triglyceride levels correlated with the rate of change in insulin secretion over two years in prediabetic subjects**. *Annals of nutrition & metabolism* 2014, **64**(1):38-43.

**Tables and Figure Legend**

**Table 1:** Baseline characteristics of the study population according to the TyG quartile groups.

**Table 2:** Baseline characteristics of the study population according to the TG/HDL-C ratio quartile groups.

**Table 3:** Multivariable Cox regression analysis of the association between the TyG index and TG/HDL-C ratio with the risk of diabetes in patients with prediabetes.

**Table 4:** Stratified analyses of the associations between the TyG index or TG/HDL-C ratio and the risk of diabetes in patients with prediabetes.

**Figure 1:** flowchart of study participants selection.

**Figure 2:** Dose-response association of the TyG index or TG/HDL-c ratio with the risk of diabetes in participants with prediabetes. The solid line represents the nonlinear dose-response, and the dotted lines represent the 95% confidence interval.

**Figure 3:** Receiver operating characteristic curves for the TyG index or TG/HDL-c ratio in predicting incident diabetes. Adjusted for age, sex, SBP, DBP, HDL-C, ALT, AST, BUN, Cr, BMI, family history of diabetes, smoking status, and drinking status. ROC: Receiver operating characteristic; AUC: area under the curve.

**Supporting information**

**Table S1:** Diagnostic for collinearity between the TyG index or TG/HDL-C ratio and other covariates when the risk of diabetes is the dependent variable.

**Table S2:** Areas under the receiver operating characteristic curves for the TyG index and TG/HDL-C ratio in identifying diabetes.

**Table S3:** Sensitivity analysis.

**Fig S1:** Histograms showing the population distribution of the TyG index and TG/HDL_C ratio.

**Fig S2**: Schoenfeld residual plot. (A) Schoenfeld residual plot of the TyG index changes over time with prediabetes to diabetes as the dependent variable; (B) Schoenfeld residual plot of the TG/HDL_C ratio changes over time with prediabetes to diabetes as the dependent variable. The *P*-value of Schoenfeld Residuals test result is larger than 0.05, indicating that the TyG index and TG/HDL_C ratio are not time dependent variables and can be analyzed by the Cox Proportional Hazards Model.
